# Heme-binding protein CYB5D1 couples intraflagellar redox to calcium signaling for coordinated flagellar beating

**DOI:** 10.1073/pnas.2535939123

**Published:** 2026-08-19

**Authors:** Yiwen Lin, Lijuan Zhao, Gai Liu, Xuan Deng, Stephen M. King, Kaiyao Huang

**Affiliations:** ^a^ Key Laboratory of Algal Biology, Institute of Hydrobiology, Chinese Academy of Sciences, Wuhan 430072, China; ^b^ College of Life Sciences, University of Chinese Academy of Sciences, Beijing 100039, China; ^c^ School of Life Sciences, Zhengzhou University, Zhengzhou 450001, China; ^d^ Department of Molecular Biology and Biophysics, University of Connecticut Health Center, Farmington, CT 06030-3305

**Keywords:** *Chlamydomonas*, cilia, motility, redox, Ca^2+^ spikes

## Abstract

Coordinated ciliary/flagellar beating requires precise spatiotemporal regulation of molecular motors such as dyneins, yet the molecular mechanisms governing ciliary synchrony remain poorly understood. Here, we demonstrate that a heme-binding axonemal protein CYB5D1 functions as a redox-sensitive switch that controls flagellar beating coordination by regulating Ca^2+^ dynamics. Both the D58G point mutation, which abolishes heme-binding activity, and the complete loss of CYB5D1 lead to a reduction in the flagellar redox potential. More importantly, the hyperreductive intraflagellar redox shift in the *cyb5d1* mutant increases *cis*-flagellar Ca^2+^ spike frequency and amplitude, similar to reductive treatment of wild-type flagella, resulting in the loss of flagellar beating coordination. Interestingly, oxidative treatments induced synchronized Ca^2+^ spikes across both *cis*- and *trans*-flagella of *cyb5d1* and increased flagellar beating coordination. In addition, loss of CYB5D1 raised the intraflagellar Ca^2+^ pool. These results indicate that CYB5D1 links redox sensing to Ca^2+^ signaling in ciliary coordination and reveal how the two flagella of the same cell achieve synchronized beating through redox-gated Ca^2+^ dynamics. Furthermore, CYB5D1 loss impairs gliding motility by dysregulating Ca^2+^ spiking specifically in the leading flagellum, extending the redox-Ca^2+^ regulatory axis to surface-associated flagellar behaviors. Given the evolutionary conservation of both CYB5D1 and the redox-Ca^2+^ signaling axis, this mechanism likely regulates ciliary function across eukaryotes, with implications for understanding ciliopathies and respiratory diseases.

## Significance

In this study, we focused on CYB5D1, an evolutionarily conserved, heme-binding axonemal protein that functions as a redox-sensitive switch. This protein converts redox dynamics into Ca^2+^-mediated mechanical dominance switching, representing a molecular mechanism linking these pathways. These findings establish redox-calcium crosstalk as a previously unrecognized regulatory axis in ciliary motility, opening avenues for mechanistic dissection and therapeutic intervention in ciliopathies including primary ciliary dyskinesia.

Cilia/flagella, evolutionarily conserved microtubule-based organelles extending from the surface of most eukaryotic cells, are essential for motility, environmental sensing, and secretion (1–5). Coordinated ciliary beating is the principal driver of mucociliary clearance in the human airway; it also propels cerebrospinal fluid within the ventricles and mediates ovum transport through the fallopian tubes, making it essential to normal function of the nervous and reproductive systems (6–8). Recent studies suggest precise ciliary beating coordination is achieved via two primary mechanisms: hydrodynamic coupling (sensing fluid flows of individual flagella) and basal coupling (structural linkage through basal connective fibers enabling mechanical force transmission) (9–12). While hydrodynamic interactions alone can drive synchronization in isolated flagella systems (e.g., *Volvox* flagellar pairs and bead-spring models) (9, 10, 13–15), unicellular organisms like *Chlamydomonas* exhibit more complex interflagellar coordination patterns, including in-phase, anti-phase, and phase-slip states (11, 16–18). Emerging evidence suggests intrinsic ciliary regulatory mechanisms may actively control flagellar synchrony beyond purely physical interactions (12, 19–21).

Ciliary beating is powered by axonemal dyneins. The generation of an effective stroke requires synchronous activation of specific dynein subsets—achieved through AAA^+^ ATPase-mediated conversion of ATP binding, hydrolysis, and product release into sliding between doublet microtubules (22–24). This sliding must be spatially restricted to generate bending—a process explained by the “switch point” hypothesis in which the dyneins on opposite axonemal sides are in alternate activity states (22). Dynein activity is precisely modulated through integrated biochemical and mechanical pathways. Biochemically, Ca^2+^ fluxes, phosphorylation cascades, and redox states dynamically tune motor function (25). Mechanistically, the intrinsic coordinated activity of thousands of axonemal dyneins drives bending of the doublet microtubules to generate the ciliary waveform, while extrinsic factors like hydrodynamic coupling between neighboring cilia (9, 10) and basal body connective structures (11, 12), as well as axonemal orientation and basal body polarity, synchronize motility (26). Complementing extrinsic mechanical coupling, Ca^2+^ and redox serve as local, fast-diffusing regulators that fine-tune dynein activity with high spatiotemporal precision and may play an important role in coordinating ciliary beating.

Calcium (Ca^2+^) is a critical second messenger in cilia/flagella, and acts as a central orchestrator of ciliary motility (11, 27, 28). First, submicromolar Ca^2+^ concentrations establish *cis*–*trans* flagellar dominance in *Chlamydomonas*—the *cis*-flagellum (closer to the eyespot) dominates steering at <10^−8^ M free Ca^2+^, while elevated concentrations (10^−7^ to 10^−6^ M) shift dominance to the *trans*-flagellum (farther from the eyespot) (29), enabling precise phototactic navigation (30). Moreover, Ca^2+^ modulates waveform asymmetry, a critical feature for chemotactic behaviors. For example, in sea urchin sperm, Ca^2+^ spikes transform symmetric beating into asymmetric waveforms, allowing sharp turning during egg-seeking navigation (20, 31–33). In contrast, *Chlamydomonas* flagella exhibit the opposite response: increasing intraflagellar Ca^2+^ converts asymmetric beating into symmetric waveforms (resulting in transient backward swimming), a distinction that reflects evolutionary divergence in the calcium sensors regulating dynein activity (34, 35), likely as an adaptation to different flagellar numbers (two in *Chlamydomonas* vs. one in sperm). At the molecular level, the cellular effects of Ca^2+^ are mediated through dedicated sensor–effector systems. Specifically, centrin-bound inner-arm dyneins accelerate microtubule sliding upon Ca^2+^ binding via their EF-hand motifs (36–38), while the LC4 calmodulin-like protein associated with the outer arm γ dynein heavy chain potentially links Ca^2+^ fluctuations (Kd ~3 × 10^−5^ M) to waveform reversal during *Chlamydomonas* photophobic responses (39–41). Collectively, these molecular adaptations regulated by Ca^2+^ ensure motility plasticity across physiological contexts.

Emerging evidence also highlights redox regulation as a critical yet underestimated layer of ciliary control, operating in parallel with Ca^2+^ signaling (21, 42–45). In *Chlamydomonas*, loss of CYB5D1 causes a reductive shift in the flagellar redox state and uncoordinated flagellar beating; however, this defect was rescued by oxidant treatment (21). Moreover, oxidizing agent treatment reduces flagellar beat frequency, while reducing agents have little effect on the flagellar beat frequency (21, 43). Similarly, airway epithelial cilia exhibit impaired mucociliary transport under oxidative stress (46). Moreover, redox poise governs phototactic directionality, such that treatment with oxidants results in positive phototaxis, while treatment with reductants leads to negative phototaxis (21, 42, 44). These responses are mediated by redox-sensitive components within motility complexes, including the outer arm thioredoxin-like proteins (LC3/LC5) and γ-heavy chain (γ-HC), and outer dynein arm-docking complex DC3 (45, 47, 48). Notably, human orthologs (TXNDC3/6) of *Chlamydomonas* redox sensors LC3 and LC5 are linked to respiratory pathologies (49, 50), underscoring the evolutionary functional conservation of these proteins. The docking complex protein DC3 exemplifies the molecular crosstalk between the Ca^2+^ and redox signaling—its Ca^2+^-binding EF-hand motif is allosterically modulated by redox-dependent disulfide formation between adjacent cysteines (47). While the functional implications of Ca^2+^ binding to DC3 remain unclear, this dual regulation suggests integrated redox-Ca^2+^ control at the level of individual dynein motors. However, the causal relation between the redox and Ca^2+^ signaling and function of the key molecular switch is unclear.

To understand how ciliary coordination is achieved through redox and Ca^2+^ signaling at the molecular level, we investigated CYB5D1, a radial spoke protein essential for flagellar synchrony. Our analysis revealed that CYB5D1 functions as a redox-sensitive molecular switch that couples intraflagellar redox state to Ca^2+^ dynamics, establishing a hierarchical regulatory pathway for ciliary coordination. Below we present evidence for each component of this mechanism.

## Results

### Full-Length CYB5D1 Is Essential for Flagellar Coordination.

**Fig. 1.**
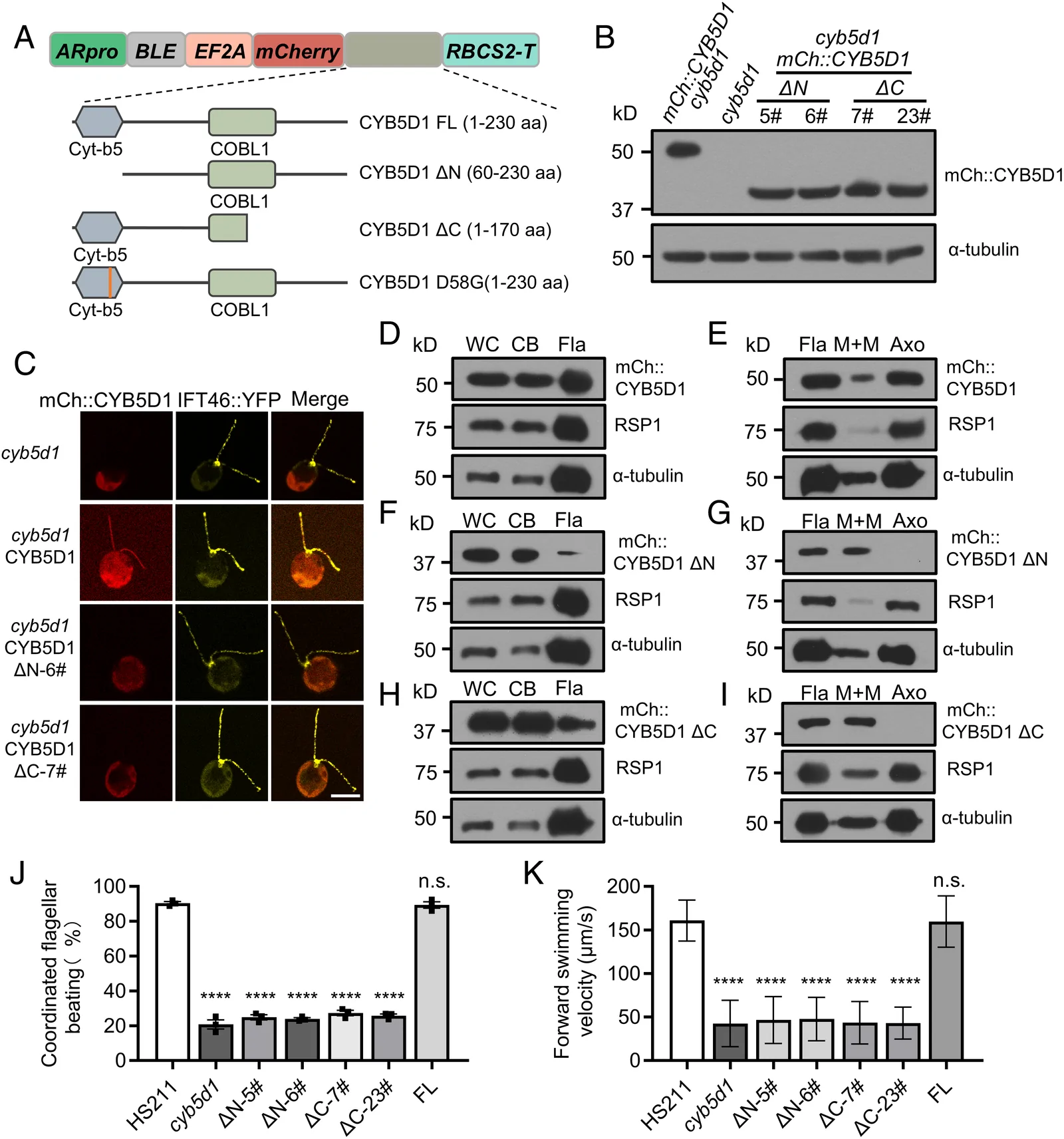
Both N- and C-terminal domains of CYB5D1 are required for flagellar localization and beating coordination. (*A*) Domain architecture of full-length, truncated, and D58G point mutant CYB5D1 constructs. (*B*) Expression of full-length and truncated proteins. (*C*) Localization of full-length and truncated proteins. (Scale bar, 5 μm.) (*D*–*I*) Subflagellar fractionation showing that axonemal localization requires both N- and C-terminal domains. N > 50, WC (whole cell), CB (cell body), Fla (flagella), M + M (membrane and matrix), and Axo (axoneme). (*J* and *K*) Quantification revealed that coordination dropped from 90.3% to below 28%, accompanied by a reduction in swimming velocity from 160.8 ± 23.3 μm/s to 40 ± 26.6 μm/s with either domain deletion. HS211 is the parental strain of *cyb5d1* mutant. Data are presented as mean ± SD. Statistical comparisons among groups were performed using one-way ANOVA with Tukey’s post hoc test. n > 50 cells per strain. Statistical significance was defined as *P* < 0.05. ****P < 0.0001.

CYB5D1 contains two conserved domains: an N-terminal heme-binding/cytochrome b5-like domain and a C-terminal cordon-bleu ubiquitin-like domain (COBL1) (21). To dissect the function of these two domains, we generated full length and two truncations of CYB5D1 (ΔN: N-terminal deletion; ΔC: C-terminal deletion) fused with mCherry fluorescent protein (Fig. 1*A*). These three fusion proteins were successfully expressed at comparable levels in the *cyb5d1* null mutant background (Fig. 1*B*). When examined by confocal microscopy, full-length mCherry::CYB5D1 (FL) exhibited a continuous signal along the flagella as did IFT46::YFP, whereas the ΔN and ΔC truncations showed no detectable mCherry fluorescence in flagella (Fig. 1*C*). Furthermore, flagellar fractionation assays demonstrated that mCherry::CYB5D1-FL predominantly localized to axonemal fractions as did the radial spoke marker RSP1 (Fig. 1 *D* and *E*). In contrast, less than one-fifth of ΔN and ΔC truncated forms of CYB5D1 entered the flagella; these were completely retained in membrane-matrix fractions and were absent from axonemes (Fig. 1 *F*–*I*).

Consistent with this mislocalization, both truncation mutants exhibited severe motility defects. Coordinated beating—defined here as in-phase (IP) synchrony, with anti-phase (AP) and slip states classified as uncoordinated (21). We observed that 90.3% ± 1.3% of the parental strain cells exhibited coordinated flagellar beating (Fig. 1*J*), and maintained prolonged linear trajectories (*SI Appendix*, Fig. S1*A*) with an average forward swimming velocity of 160.8 ± 23.3 μm/s (Fig. 1*K*). In marked contrast, both the *cyb5d1* mutant and *cyb5d1* mutant rescued with constructs harboring N-terminal (ΔN) or C-terminal (ΔC) truncations exhibited noticeable motility defects; 74.4% ± 2.4% of cells displayed uncoordinated beating patterns and constrained oscillatory movements (“rocking”) which resulted in truncated irregular swimming paths (*SI Appendix*, Fig. S1*A*) with a forward swimming velocity reduced to 40 ± 26.6 μm/s (Fig. 1*K*). Similarities in flagellar beat frequency and progressive swimming capacity were observed in the *cyb5d1* mutant and rescued strains harboring N-terminal (ΔN) or C-terminal (ΔC) CYB5D1 deletions (*SI Appendix*, Fig. S1 *B* and *C*); notably, neither truncation affected the percentage of flagellated cells or flagellar length (*SI Appendix*, Fig. S1 *D* and *E*). These results demonstrate that both N- and C-terminal domains of CYB5D1 are required for its flagellar targeting and integration into radial spoke complexes, and CYB5D1 is essential for flagellar beating coordination.

### Molecular Basis of CYB5D1 in Redox Sensing.

**Fig. 2.**
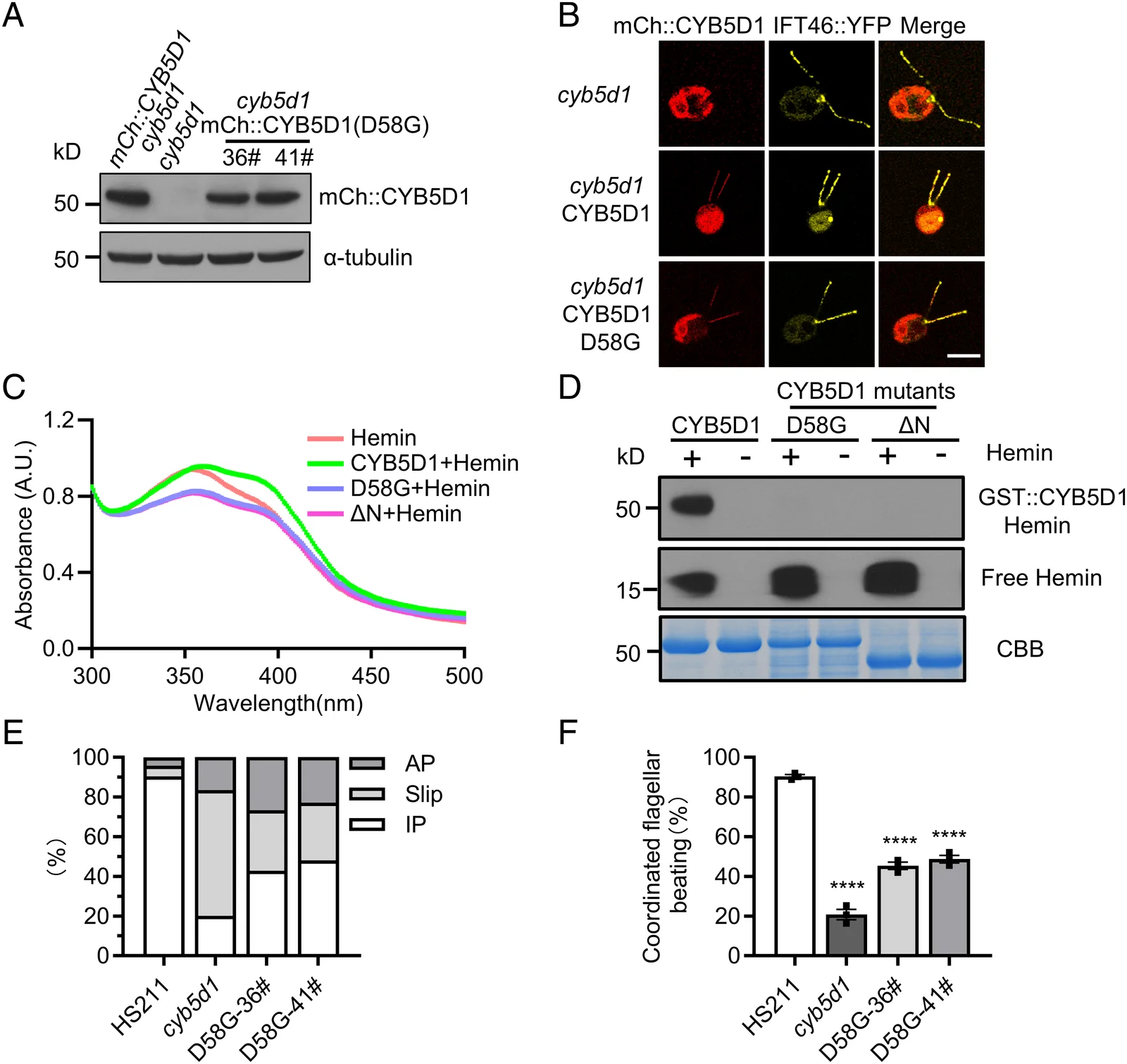
The D58 residue of CYB5D1 is critical for heme binding and flagellar beating coordination. (*A* and *B*) Expression and localization of D58G point mutations in CYB5D1 in the *cyb5d1* mutant strain. (Scale bar, 5 μm.) (*C*) Spectroscopic analysis revealed that the D58G mutation eliminates heme binding (402 nm peak absent), while all curves show a similar decay for values above 500 nm. (*D*) Detection of heme-promoted peroxidase activity using purified GST::CYB5D1. The *Top* panel shows the peroxidase activity of GST::CYB5D1–hemin complex, the *Middle* panel shows the peroxidase activity of free Hemin, and the *Bottom* panel shows Coomassie Brilliant Blue staining. Peroxidase activity assay reveals a signal only when CYB5D1 is mixed with hemin and confirms loss of heme binding in the D58G mutant. This establishes CYB5D1 as a heme-dependent redox sensor. (*E* and *F*) The D58G mutation decreases flagellar coordination from 90.3% to below 48.7%. ****P < 0.0001, n = 3 independent experiments.

Homology alignment of CYB5D1 with human CYB5D2 suggested that D58 near the N terminus is conserved and may mediate heme interaction, analogous to its role in CYB5D2 (21, 51). This hypothesis was further supported by AlphaFold 3 modeling (52); the heme-binding pocket is constricted upon D58G substitution (*SI Appendix*, Fig. S2 *A* and *B*). To investigate the function of this residue, we generated a CYB5D1(D58G) construct using the strategy in Fig. 1*A* and obtained a *cyb5d1* strain expressing mCherry::CYB5D1(D58G) (Fig. 2*A*). The mCherry-tagged CYB5D1(D58G) was localized in the flagella as is wild-type (WT) CYB5D1 (Fig. 2*B*), indicating that the D58G mutation did not change the flagellar targeting of CYB5D1.

To determine whether the D58 residue of CYB5D1 is essential for heme-binding, GST-tagged recombinant proteins CYB5D1, CYB5D1(D58G), CYB5D1(ΔN) were expressed in *Escherichia coli* BL21 and purified using glutathione-agarose (*SI Appendix*, Fig. S3). To test hemin binding, we exploited the characteristic spectral shift (382→402 nm) exhibited by CYB5D1–hemin complexes (21), measuring absorbance after mixing 5 μg of each purified protein with 5 nM hemin. Only the mixture of GST::CYB5D1 and heme caused spectral shift (Fig. 2*C*), indicating that the hemin–protein complex formed. In addition, a strong signal for peroxidase activity emerged in the mixture of GST::CYB5D1 and hemin, whereas no peroxidase activity was observed in D58G or ΔN mutants with hemin (Fig. 2*D*, *Upper* panel), confirming that D58 is necessary for functional heme interactions. These findings demonstrate that the D58 residue is indispensable for the heme-binding capacity of CYB5D1.

Consistent with this loss of heme-binding activity, severe flagellar beating coordination defects were observed in the *cyb5d1* CYB5D1(D58G) mutant. In the parental strain HS211, Slip and AP states accounted for only 4% and 5% of beat cycles, respectively. In contrast, these values increased to 30% and 25% in the *cyb5d1* CYB5D1(D58G) mutant (Fig. 2*E*). Furthermore, coordinated beating decreased from 90.3% ± 1.3% in HS211 to 47.0% ± 3.1% (Fig. 2*F*), accompanied by a 26.82% reduction in forward swimming cells (62.2% ± 4.3% vs. 89.0% ± 1.4% for HS211; *SI Appendix*, Fig. S1*C*). Therefore, swimming trajectories shifted from linear to spiral patterns, with beat frequency increasing marginally from 59.2 ± 1.9 to 62.6 ± 2.9 Hz (*SI Appendix*, Fig. S1 *A* and *B*). As expected, flagellar length and the percentage of flagellated cells remained comparable to WT (*SI Appendix*, Fig. S1 *D* and *E*). Together, these results establish that the D58 residue of CYB5D1 is critical for both heme binding and coordinated flagellar beating.

### CYB5D1 Maintains Intraflagellar Redox Homeostasis.

**Fig. 3.**
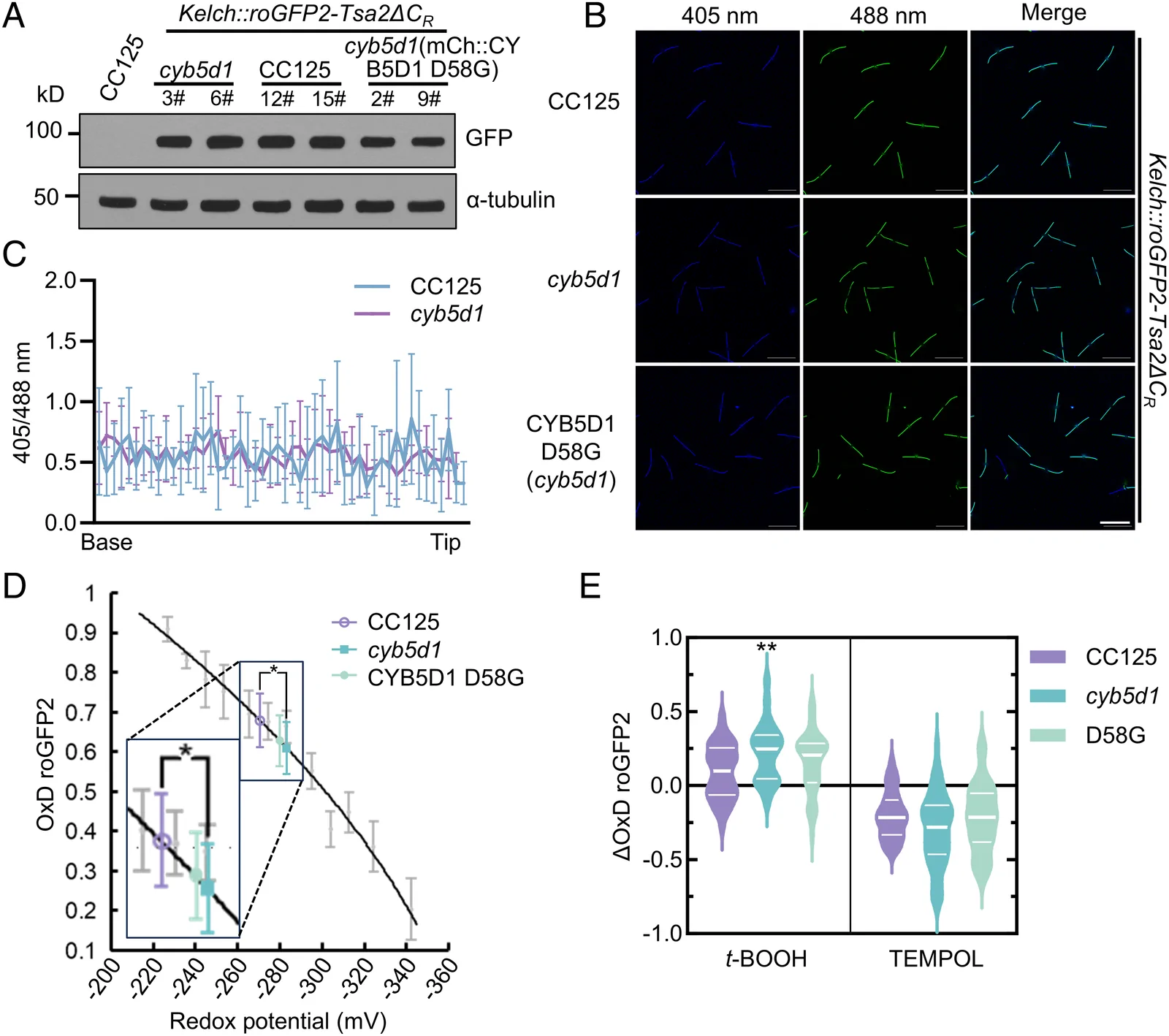
The deletion or the D58G point mutation of CYB5D1 reduced redox potential within the flagella. (*A* and *B*) Expression and localization of Kelch::roGFP2-Tsa2ΔC_R_. (Scale bar, 10 μm.) (*C*) The fluorescence signal of Kelch::roGFP2-Tsa2ΔC_R_ along the flagella in WT and mutant strains was analyzed statistically. (*D*) Oxidized roGFP2 ratio derived from a standard curve (calculated per ref. 58, r^2^ = 0.976). (*E*) Redox potential changes in flagella upon *t*-BOOH (oxidant) or TEMPOL (reductant) treatment. Statistical analysis was performed using two-way ANOVA with Tukey’s post hoc test to assess the effects of strain and treatment. N > 45, **P < 0.01; Comparisons without annotation showed no statistical evidence of a difference (*P* > 0.05).

Loss of CYB5D1 induces a hyperreductive state in the flagellum, as read out by the redox-sensitive reporter LC3 (21). To quantify flagellar redox potential in *cyb5d1* in real time, we employed the roGFP2–Tsa2ΔC_R_ biosensor, in which a yeast peroxiredoxin (Tsa2) is fused to roGFP2 to improve oxidative robustness, reduce susceptibility to hyperoxidation, and maintain pH stability across the range pH 6.0 to 8.5 (53–55). The roGFP2-Tsa2ΔC_R_ was fused to the first Kelch repeat domain of the outer arm dynein α-heavy chain to create a flagellum-anchored reporter. This Kelch repeat domain represents the N-terminal 333-amino acids of the α-heavy chain and forms a β-propeller structure that interacts directly with the outer arm β heavy chain yielding a 24-nm periodicity in axoneme (*SI Appendix*, Fig. S4*A*) (56, 57). The Kelch::roGFP2-Tsa2ΔC_R_ was transformed into WT strain CC125 and the *cyb5d1* mutant, and was also introduced into *cyb5d1* CYB5D1(D58G) mutant through a genetic cross with CC124 (mt-) to remove IFT46::YFP in the mCh::CYB5D1 (D58G) (*cyb5d1*) strain, and maintain an isogenic background (*SI Appendix*, Fig. S5 *A* and *B*). Immunoblot analysis showed that the expression level of Kelch::roGFP2-Tsa2ΔC_R_ is similar in CC125, *cyb5d1* and *cyb5d1* CYB5D1(D58G) cells (Fig. 3*A*). To quantify the signal of Kelch::roGFP2-Tsa2ΔC_R_ in flagella, cells were observed using STED (Stimulated Emission Depletion) ultra-high-resolution microscopy. The uniform emission fluorescence distribution along flagella was observed at both 405 nm (oxidized state) and 488 nm (reduced state) excitation wavelengths (Fig. 3*B*), confirming uniform redox sensor localization of Kelch::roGFP2-Tsa2ΔC_R_. Using the 405/488 nm ratio for nanometer-scale redox potential mapping along the flagellar axoneme, we found that redox potential was uniform along the length of the flagella in both WT and the *cyb5d1* mutant, with no *cis*–*trans* difference (Fig. 3*C* and *SI Appendix*, Fig. S4*B*). In contrast, the *cyb5d1* CYB5D1(D58G) mutant exhibited a slightly lower ratio in the *cis*-flagellum compared to the *trans*-flagellum (*SI Appendix*, Fig. S4*B*).

To determine the quantitative relationship between the roGFP2-Tsa2ΔC_R_ fluorescence ratio and redox potential for use as a calibration curve, isolated flagella were demembranated and sequentially equilibrated in redox-calibrated buffers according to the method of Sugiura et al. (59) (*SI Appendix*, Fig. S4*C* and Table S1). Flagellar fluorescence was measured in each buffer condition, and the degree of roGFP2 oxidation [OxD, (55, 58)] was fitted to the Nernst equation to derive the corresponding redox potential (Fig. 3*D*). To monitor the temporal variability, time-lapse imaging of flagella over 60-s intervals was recorded and compared; we found that OxD was relatively stable during the 60-s observation (*SI Appendix*, Fig. S6 *A*–*C*). The flagellar redox potential of CC125 is −269.56 ± 12.53 mV, that of *cyb5d1* is −282.31 ± 9.37 mV, and that of *cyb5d1* CYB5D1(D58G) is −279.06 ± 11.22 mV. The intraflagellar redox potential in *cyb5d1* and *cyb5d1* CYB5D1(D58G) was decreased compared to that in WT CC125 (Fig. 3*D*), which is negatively correlated with their heme-binding activity, suggesting the important role of CYB5D1 in maintaining the homeostasis of redox potential in flagella.

When the cells were incubated in 0.4 mM *t*-BOOH (tert-butyl hydroperoxide; an oxidizing agent), the OxD (roGFP2 oxidation ratio) increased correspondingly. The flagellar OxD of CC125 increased by 0.094 (ΔOxD) (Fig. 3*E*), and *cyb5d1* mutants exhibited a ΔOxD of 0.228, which was 2.42-fold higher than the WT ΔOxD (one-sided unpaired *t* test, *P* < 0.01), indicating that CYB5D1 deficiency impairs oxidative stress buffering capacity. Notably, the *cyb5d1* (D58G) point mutant showed an intermediate ΔOxD of 0.162, which was statistically indistinguishable from either WT or *cyb5d1* in two-sided post-hoc comparisons (*P* > 0.05). Following 40 mM TEMPOL (4-hydroxy-2,2,6,6-tetramethylpiperidine 1-oxyl; a reducing agent) treatment, all strains showed decreased OxD relative to their respective untreated controls (WT: −0.200, *cyb5d1*: −0.292, D58G: −0.212; one-sided paired *t* test, all *P* < 0.0001). The similar tendencies observed imply that CYB5D1 is also involved in reductive homeostasis. These findings align with our previous report that CYB5D1 deficiency induces a hyperreductive state in flagella (21), further demonstrating the role of interaction of CYB5D1 with hemin in maintaining intraflagellar redox homeostasis in *Chlamydomonas reinhardtii*.

To test whether this reductive shift is a general consequence of motility defects, the redox sensor was also introduced into a panel of well characterized axonemal mutants: *pf14* (radial spoke-defective), and *oda11, oda4-s7, oda2-t*, and *oda1* (outer dynein arm-defective) (*SI Appendix*, Fig. S7 *A* and *B*). The flagella of radial spoke mutant *pf14* showed a slight increase in oxidation, whereas the flagella of all outer dynein arm mutants displayed reduced OxD values (more reduced), with calculated redox potentials ranging from −263.45 ± 14.08 mV (*pf14*) to −288.51 ± 21.96 mV (*oda1*), compared to −269.56 ± 12.53 mV in WT CC125 (*SI Appendix*, Fig. S7 *C* and *D*). These results indicate that the reductive shift is not an inevitable consequence of impaired motility. CYB5D1 may serve as a pivotal node whose function is distinct from that of other axonemal proteins.

### Redox Functions Upstream of Ca^2+^ in Regulating Flagellar Beating Coordination.

**Fig. 4.**
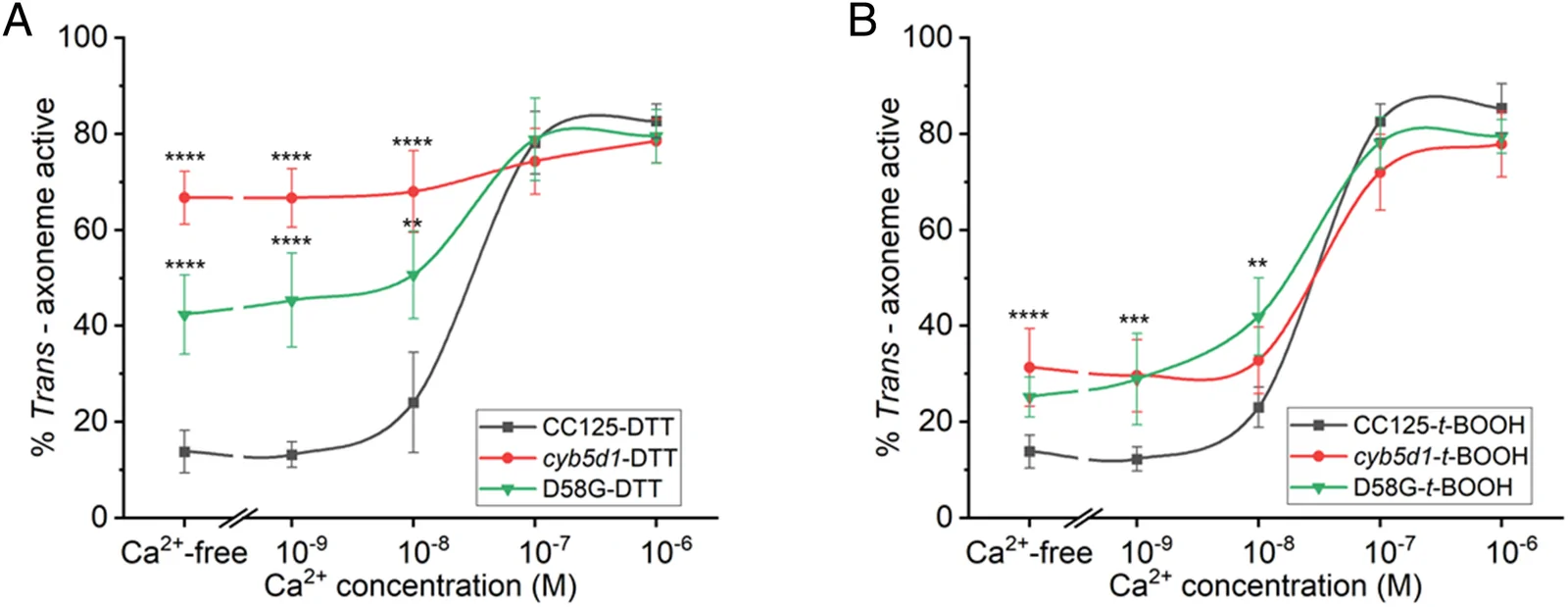
Redox functions upstream of calcium in regulating flagellar beating coordination in *Chlamydomonas*. (*A*) Axonemal dominance under different concentrations of Ca^2+^ + DTT. (*B*) Axonemal dominance under different concentrations of Ca^2+^ + *t*-BOOH. Data are presented as mean ± SD. Statistical significance was assessed using two-way ANOVA with Tukey’s post-hoc test (α = 0.05). ****P < 0.0001. Sample sizes: N > 450 cells per condition.

To determine whether changes in the redox state have an effect on flagellar dominance which is Ca^2+^ concentration-dependent, WT (CC125), *cyb5d1* and *cyb5d1* CYB5D1(D58G) cells were demembranated and reactivated in standard reactivation solutions containing 1 mM DTT, as originally described (29) and used in subsequent studies (21, 30). Ca^2+^-dependent enhancement of *trans*-flagellar dominance was observed in WT (CC125), whereas the *trans*-axoneme constantly showed dominance in *cyb5d1* cell models regardless of changes in the Ca^2+^ concentration (Fig. 4*A*) (21). *cyb5d1* CYB5D1(D58G) cells also exhibited Ca^2+^-dependent enhancement of *trans*-flagellar dominance, but the *cis*-flagellar dominance in this mutant at low Ca^2+^ concentrations was more attenuated compared to WT cells (Fig. 4*A*), indicating a direct functional link between CYB5D1 and flagellar dominance.

To further test the role of redox state in flagellar dominance, we substituted the reductant DTT with the oxidant *t*-BOOH in the reactivation solutions. This redox transition did not alter the flagellar dominance of WT cells (Fig. 4 *A* and *B*). Conversely, it recovered *cis*-flagellar dominance in both the *cyb5d1* mutant and the *cyb5d1* CYB5D1(D58G) point mutant. When oxidizing conditions were gradually increased, the extent of recovery increased progressively (*SI Appendix*, Fig. S8*A*). These results indicate that redox state may directly modulate Ca^2+^ signaling in flagellar beating coordination.

To investigate the role of Ca^2+^ in flagellar beating coordination and to assess whether a deficiency in CYB5D1 has an effect on this process, cells were resuspended in solutions with various buffered Ca^2+^ concentrations. WT CC125 cells exhibited Ca^2+^-dependent enhancement in both flagellar coordination and swimming speed as extracellular Ca^2+^ concentrations increased from 10^−9^ M to 10^−4^ M (*SI Appendix*, Fig. S8 *B* and *C*). Notably, even in Ca^2+^-free conditions, WT cells retained 50% coordinated flagellar beating and maintained a swimming speed of ~80 μm/s. In contrast, the beating coordination of *cyb5d1* and the point mutant *cyb5d1* CYB5D1(D58G) lost the Ca^2+^ dependence of flagellar beating coordination across a broad concentration range (10^−9^ to 10^−4^ M), while their swimming speeds remained largely constant (*SI Appendix*, Fig. S8 *B* and *C*). These findings collectively demonstrate that Ca^2+^ acts as a downstream effector of redox changes mediated by CYB5D1 to regulate flagellar dominance and coordinate flagellar beating.

### Redox Modulates Asymmetric Ca^2+^ Spikes in *Chlamydomonas* Flagella.

To directly visualize the dynamic changes in Ca^2+^ levels within flagella and confirm that redox functions upstream of Ca^2+^, we selected the ratiometric biosensor MatryoshCaMP6s (60). To enhance the expression of this Ca^2+^ sensor, we optimized the coding sequence and inserted three introns from the nuclear RuBisCO small subunit gene RBCS2 into the coding sequence (61). Subsequently, the MatryoshCaMP6s was inserted at the C-terminus of the Kelch repeat domain of the outer arm α-heavy chain (*SI Appendix*, Fig. S9*A*), and expressed in CC125, *cyb5d1* and *cyb5d1* CYB5D1(D58G) strains. Immunoblot analysis showed the 110 kDa fusion protein is expressed at similar levels in all these strains (*SI Appendix*, Fig. S9*B*). Then, the flagellar fluorescence was recorded using STED microscopy. We found that the GCaMP6s and LSSmOrange fluorescent signals from MatryoshCaMP6s were distributed “uniformly” along the flagella (Ca^2+^ spikes sometimes occurred) (*SI Appendix*, Fig. S9*C*).

To verify that axonemal Kelch::MatryoshCaMP6s signals scale quantitatively with free Ca^2+^, we measured reporter fluorescence in isolated axonemes after permeabilization of the flagellar membrane with 0.2% NP-40, thereby ensuring unrestricted Ca^2+^ access (*SI Appendix*, Fig. S9*D*). Axonemes were then incubated in a series of Ca^2+^-buffers spanning physiological concentrations from 0 μM to 39 μM. GCaMP6s fluorescence showed a sigmoidal curve with respect to free Ca^2+^ concentration, while the fluorescence intensity of LSSmOrange was almost unaffected by the change in Ca^2+^ concentration (*SI Appendix*, Fig. S9*E*). The blue dotted line in *SI Appendix*, Fig. S9*E* represents the calibration curve for quantitatively measured flagellar Ca^2+^ based on Kelch::MatryoshCaMP6s fluorescence (r^2^ = 0.98). This optimized MatryoshCaMP6s system is capable of precise Ca^2+^ imaging in flagella of *Chlamydomonas*.

**Fig. 5.**
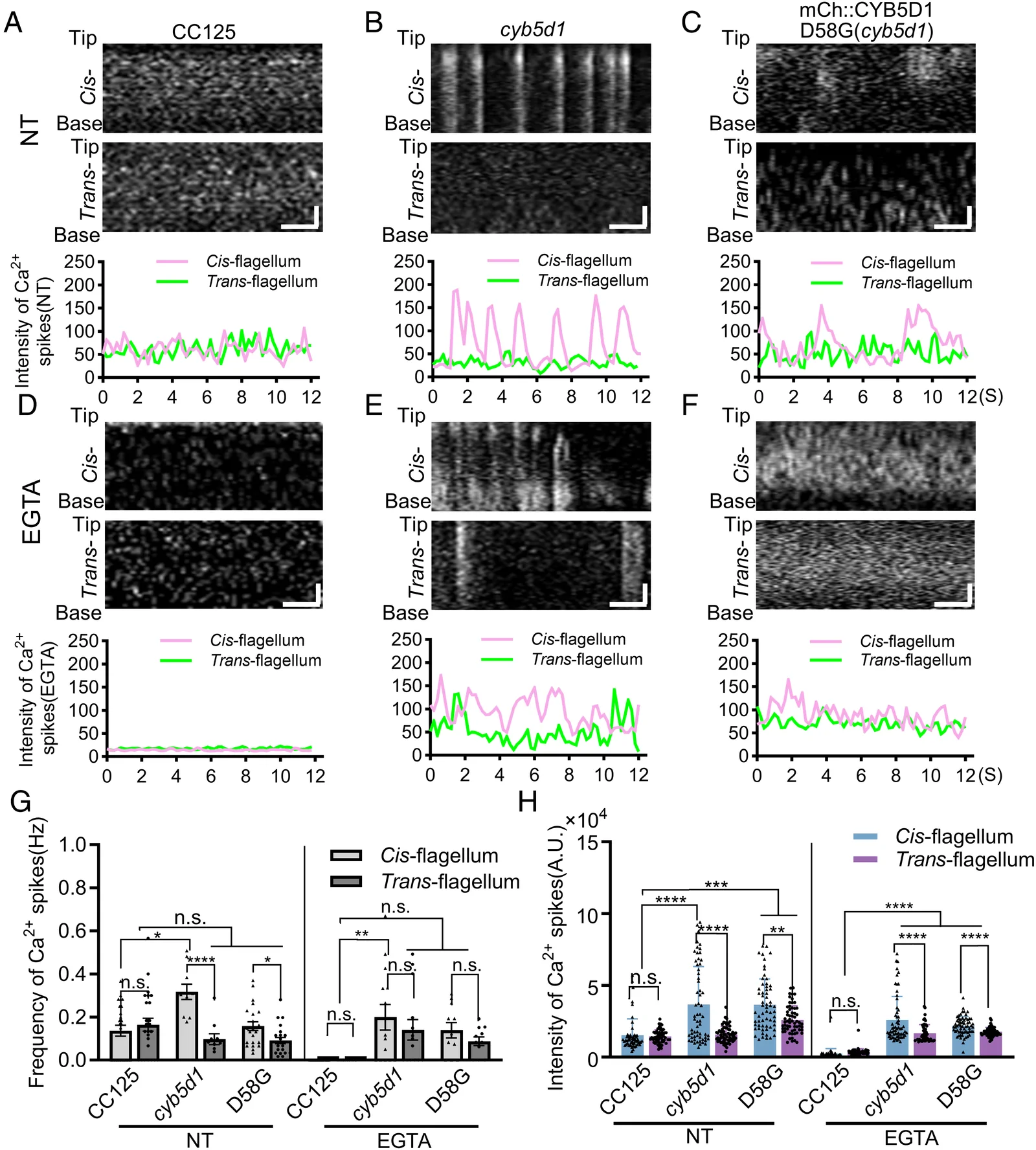
Reduced redox potential enhanced Ca^2+^ spikes in *cis*-flagella. (*A–C*) Real-time STED imaging of Ca^2+^ dynamics using MatryoshCaMP6s in WT and mutant strains. CYB5D1 deletion induces a higher Ca^2+^ spike frequency in the *cis*-flagellum (0.32 Hz) than in the *trans*-flagellum (<0.1 Hz), and the intensity increases by 1.32 times. Point mutant shows an intermediate phenotype. Horizontal scale: 2 s; vertical scale: 2 μm. (*D–F*) Ca^2+^ spikes in *cis/trans*-flagella after treated with 1 mM EGTA (~150 nM free Ca^2+^). Horizontal scale: 2 s; vertical scale: 2 μm. (*G* and *H*) Quantification of spike frequency and intensity. Data are presented as mean ± SD; n > 11 cells per strain; two-way ANOVA with Šidák’s test. ****P < 0.0001.

To continuously monitor Ca^2+^ dynamics in flagella, *Chlamydomonas* cells were immobilized in 1% agarose chambers and imaged via STED microscopy (Movies S1–S6). The spatiotemporal Ca^2+^ dynamics in flagella were analyzed using kymographs. In WT *Chlamydomonas* (CC125) cells, flagellar Ca^2+^ spikes exhibited stable, low-frequency oscillations (~0.15 Hz) with no *cis*–*trans* flagellar difference in either the frequency or intensity of the Ca^2+^ spikes (Fig. 5 *A*, *G,* and *H* and Movie S1, *Left* panel).

In the absence of CYB5D1, the *cis*-flagellum exhibited a markedly higher Ca^2+^ spike frequency (0.32 Hz) compared to the *trans*-flagellum (<0.1 Hz) (Fig. 5 *B* and *G*), and also displayed a greater Ca^2+^ spike magnitude (Fig. 5 *B* and *H*). In the point mutant strain *cyb5d1* CYB5D1(D58G), *cis*-flagellar Ca^2+^ spike frequency remained elevated relative to the *trans*-flagellum, although their frequency was reduced compared to *cyb5d1* (Fig. 5 *B*, *C,* and *H*). Notably, the *cyb5d1* CYB5D1(D58G) mutant exhibited slower decay of Ca^2+^ signal intensity compared to *cyb5d1* mutants (Movie S2, *Middle* and *Right* panels), while the peak spike intensity remained comparable (Fig. 5*H*). This indicates that changes in the flagellar redox potential of *cyb5d1* may modulate Ca^2+^ dynamics within the flagellum directly, and that the D58G mutation may impair signal termination specifically.

To determine the source of calcium ions for the spike events, the cells were treated with 1 mM EGTA to chelate extracellular free Ca^2+^, reducing its concentration to cytosolic levels (~150 nM) (62) (Fig. 5 *D*–*F* and Movie S2). In WT CC125 flagella, no fluorescence fluctuations were observed after 1-min EGTA treatment (Fig. 5*D* and Movie S2, *Left* panel), indicating that extracellular Ca^2+^ is the primary spike source, as previously reported (62). Surprisingly, *cyb5d1* mutants retained residual Ca^2+^ fluctuations under 1 mM EGTA treatment (Fig. 5*E* and Movie S2, *Middle* panel), albeit with reduced frequency and intensity compared to untreated conditions (reduced by ~1/3) (Fig. 5 *G* and *H*). EGTA treatment of point mutant *cyb5d1* CYB5D1(D58G) yielded similar phenotypes as *cyb5d1*; Ca^2+^ spikes persisted (Fig. 5*F* and Movie S2, *Right* panel) despite reduced frequency and intensity (Fig. 5 *G* and *H*), which were more attenuated compared to *cyb5d1* cells. These data suggest that changes in the redox potential in flagella also result in a higher basal concentration of calcium ions within flagella.

### Redox Modulation Rescues Ca^2+^ Spike Patterns.

**Fig. 6.**
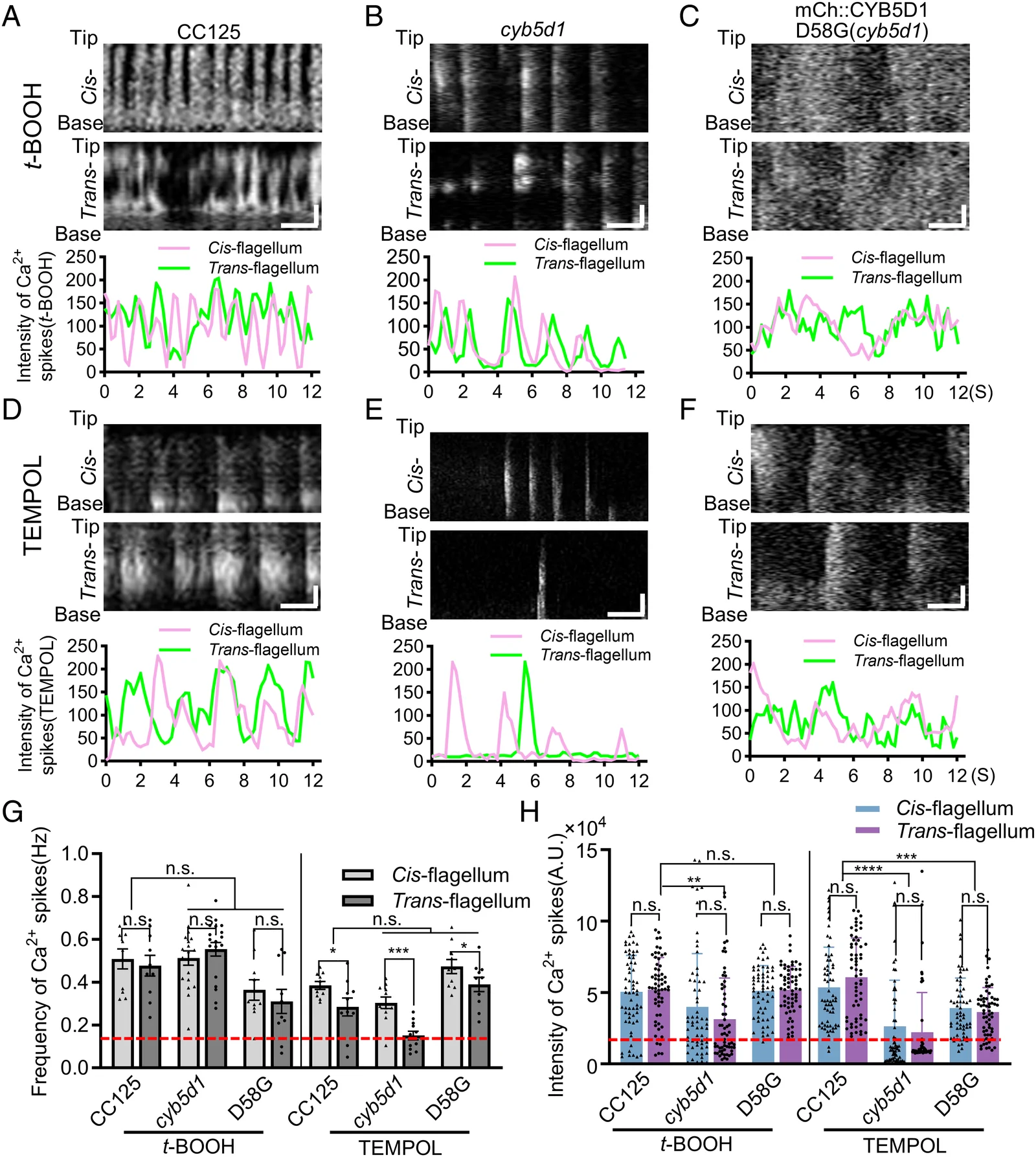
Redox state directly controls flagellar Ca^2+^ synchrony. (*A–C*) Oxidant treatment with *t*-BOOH synchronizes Ca^2+^ spikes between flagella and rescues coordination in CYB5D1 mutants. (*D–F*) Reductant treatment with TEMPOL disrupts spike synchrony in WT cells. These results demonstrate that redox signaling acts upstream of Ca^2+^ in ciliary regulation. Horizontal scale: 2 s; vertical scale: 2 μm. (*G* and *H*) Quantification shows oxidants double spike frequency and restore *cis–trans* coupling, while reductants have opposite effects. Red dashed line: Average Ca^2+^ spike frequency or intensity in untreated WT CC125 strains. Data are presented as mean ± SD; n > 11 cells per strain; two-way ANOVA with Šidák’s test. ****P < 0.0001.

To test whether flagellar Ca^2+^ spikes are regulated by redox directly, oxidizing (*t*-BOOH) or reducing (TEMPOL) agents were used to treat the cells. Strikingly, *t*-BOOH treatment of WT (CC125) cells induced highly synchronized Ca^2+^ spikes between *cis*- and *trans*-flagella (Fig. 6*A* and Movie S3, *Left* panel), with spike frequency tripling to ~0.5 Hz compared to untreated controls (0.15 Hz) (Fig. 5*G*). In the *cyb5d1* mutant, *t*-BOOH partially restored Ca^2+^ spikes in *trans*-flagella, and both flagella exhibited synchronized spikes (Fig. 6*B* and Movie S3, *Middle* panel), albeit with a slight temporal shift/delay compared to WT (Fig. 6 *A* vs. *B*). *t*-BOOH treatment of *cyb5d1* CYB5D1(D58G) restored *trans*-flagellar spikes (Fig. 6*C* and Movie S3, *Right* panel); however, these spikes exhibited profoundly slower postspike Ca^2+^ decay compared to both WT and *cyb5d1* controls (Fig. 6 *B* and *C*), which likely underlies their modestly reduced spike frequency.

We next assessed whether the reducing agent TEMPOL also modulates Ca^2+^ dynamics in flagella. In WT *Chlamydomonas* (CC125), TEMPOL treatment increased flagellar Ca^2+^ spike frequency to 0.28 to 0.38 Hz compared to untreated controls (0.15 Hz) (Fig. 6*D*), albeit less pronounced than *t*-BOOH-induced increases (Fig. 6*G*), and the *cis*–*trans* coupling of Ca^2+^ spikes was disrupted (Fig. 6*D* and *SI Appendix*, Fig. S11 and Movie S4, *Left* panel). In addition to a reduced frequency of Ca^2+^ spikes in the *trans*-flagellum (Fig. 6*G*), the prolonged duration of individual spike events resulted in marginally higher cumulative intensity in the *trans*-flagellum (Fig. 6 *D* and *H*). In the *cyb5d1* mutant, TEMPOL treatment did not alter Ca^2+^ spike patterns; both frequency and intensity of Ca^2+^ spikes remained unchanged compared to untreated *cyb5d1* cells, but the spikes decayed more rapidly (Fig. 6*E* and Movie S4, *Middle* panel). Strikingly, TEMPOL treatment of *cyb5d1* CYB5D1(D58G) amplified Ca^2+^ spikes, but decreased coordination between flagella (Fig. 6*H* and *SI Appendix*, Fig. S11 and Movie S4, *Right* panel), suggesting redox-Ca^2+^ crosstalk requires intact CYB5D1. These results establish that redox directly modulates both the magnitude and synchrony of flagellar Ca^2+^ spikes, with oxidative and reductive stresses exerting distinct regulatory effects. Taking into account our previous observation that only the oxidizing agent can rescue the beating coordination defect in *cyb5d1* (21), Ca^2+^ signaling likely serves as a critical node coupling flagellar redox to the synchronization of flagellar beating.

Given the marked redox-induced increase in Ca^2+^ spike frequency and amplitude (Fig. 6 *G* and *H*), we next asked whether these Ca^2+^ fluctuations persist when extracellular Ca^2+^ is chelated with 1 mM EGTA (Movies S5 and S6). In WT CC125 flagella, neither oxidants nor reductants induced Ca^2+^ fluctuations during EGTA treatment (*SI Appendix*, Fig. S10 *A* and *D*). This indicates the flagellar Ca^2+^ spikes are derived from extracellular Ca^2+^. In contrast, the *cyb5d1* mutant still maintained elevated intraflagellar Ca^2+^ spikes under EGTA treatment (*SI Appendix*, Fig. S10 *B* and *E*), while *cyb5d1* CYB5D1(D58G) displayed frequent low-amplitude Ca^2+^ fluctuations (*SI Appendix*, Fig. S10 *C* and *F*). These findings confirm flagellar redox also modulates intraflagellar Ca^2+^ stores, independent of extracellular Ca^2+^. Collectively, redox state governs both the magnitude and coordination of flagellar Ca^2+^ spikes, as well as the flagellar Ca^2+^ pool, with CYB5D1 serving as a critical node in this regulatory network.

### CYB5D1 Regulates Ca^2+^ Dynamics in Swimming Flagella.

To determine Ca^2+^ dynamics in swimming *Chlamydomonas* flagella, we optimized the real-time imaging strategy to overcome two significant challenges: the high flagellar beat frequency (~60 Hz) and the subdiffraction-scale flagellar diameter (~250 nm). Treatment with 10% Ficoll-400 reduced beat frequency to ~20 Hz (*SI Appendix*, Fig. S12), while 10 μm silica beads enhanced focal plane retention, enabling sustained visualization of intraflagellar Ca^2+^ fluctuations. The flagellar beating and Ca^2+^ dynamics were recorded using a Nikon AXR superresolution resonance scanning microscope (250 fps). WT (CC125) cells predominantly exhibited in-phase (IP) beating, whereas *cyb5d1* mutants showed antiphase (AP) and slip states (*SI Appendix*, Fig. S13 *B* and *C*), consistent with previous observations (Fig. 2*E*). Quantitative analysis revealed that the frequency and intensity of the Ca^2+^ signal are similar in the same flagellar beating waveforms (*SI Appendix*, Fig. S13*B*), indicating Ca^2+^ levels are related to changes in the beating waveform. The predominance of AP/slip states in *cyb5d1* mutants, indicating a relatively high concentration of free calcium ions within the flagella, likely explains their elevated baseline Ca^2+^ levels under static conditions (*SI Appendix*, Fig. S13*E*). Notably, the weighted average Ca^2+^ intensity in free-swimming WT and *cyb5d1* cells correlates well with the mean Ca^2+^ spike intensity measured in agarose-immobilized cells (*SI Appendix*, Fig. S13 *D* and *E*). Together, these complementary approaches demonstrate that although cell immobilization enabled precise resolution of individual Ca^2+^ spike events, the analysis of freely swimming cells confirms that such events faithfully reflect flagellar coordination behavior under physiological conditions. These results delineate flagellar Ca^2+^ as an important regulator for maintaining the flagellar coordinated beating.

### CYB5D1 Is also Required for Gliding Motility.

To determine whether the coordination defect of *cyb5d1* mutant extends to surface-associated behaviors such as gliding motility, which depends on intraflagellar Ca^2+^ signaling (63), we examined the gliding behavior of the mutant cells. During gliding, the leading flagellum normally plays the dominant role in generating pulling force, whereas the trailing flagellum is thought to be relatively passive (63). Both *cyb5d1* and *cyb5d1* (D58G) mutants exhibited intact surface adhesion but reduced gliding velocities (0.83 ± 0.38 μm/s and 0.95 ± 0.32 μm/s, respectively) compared to WT (1.24 ± 0.42 μm/s) (*SI Appendix*, Fig. S14 *A* and *B*). Importantly, Ca^2+^ spike frequencies in the trailing flagellum were comparable across all strains, consistent with its passive role remaining intact. In striking contrast, the leading flagellum of *cyb5d1* and *cyb5d1* (D58G) mutants exhibited markedly elevated Ca^2+^ spike frequencies (0.21 ± 0.08 Hz and 0.15 ± 0.08 Hz, respectively) compared to WT (0.03 ± 0.04 Hz), while spike intensities remained unchanged in both flagella (*SI Appendix*, Fig. S14 *C* and *D*). These results indicate that CYB5D1 also plays a role in coordinating flagellar activity during gliding. However, the increased Ca^2+^ spike frequency in the leading flagellum without a corresponding change in spike intensity (*SI Appendix*, Fig. S14*D*)—a pattern strikingly distinct from that observed in immobilized flagella—indicates that synchronized Ca^2+^ spiking between *cis*- and *trans*-flagella is a hallmark of coordinated beating. Together, these findings indicate that CYB5D1 also contributes to flagellar coordination during gliding by restraining excessive Ca^2+^ spiking in the leading flagellum.

### A Hierarchical Redox–Ca^2+^ Pathway Controls Ciliary Coordination.

**Fig. 7.**
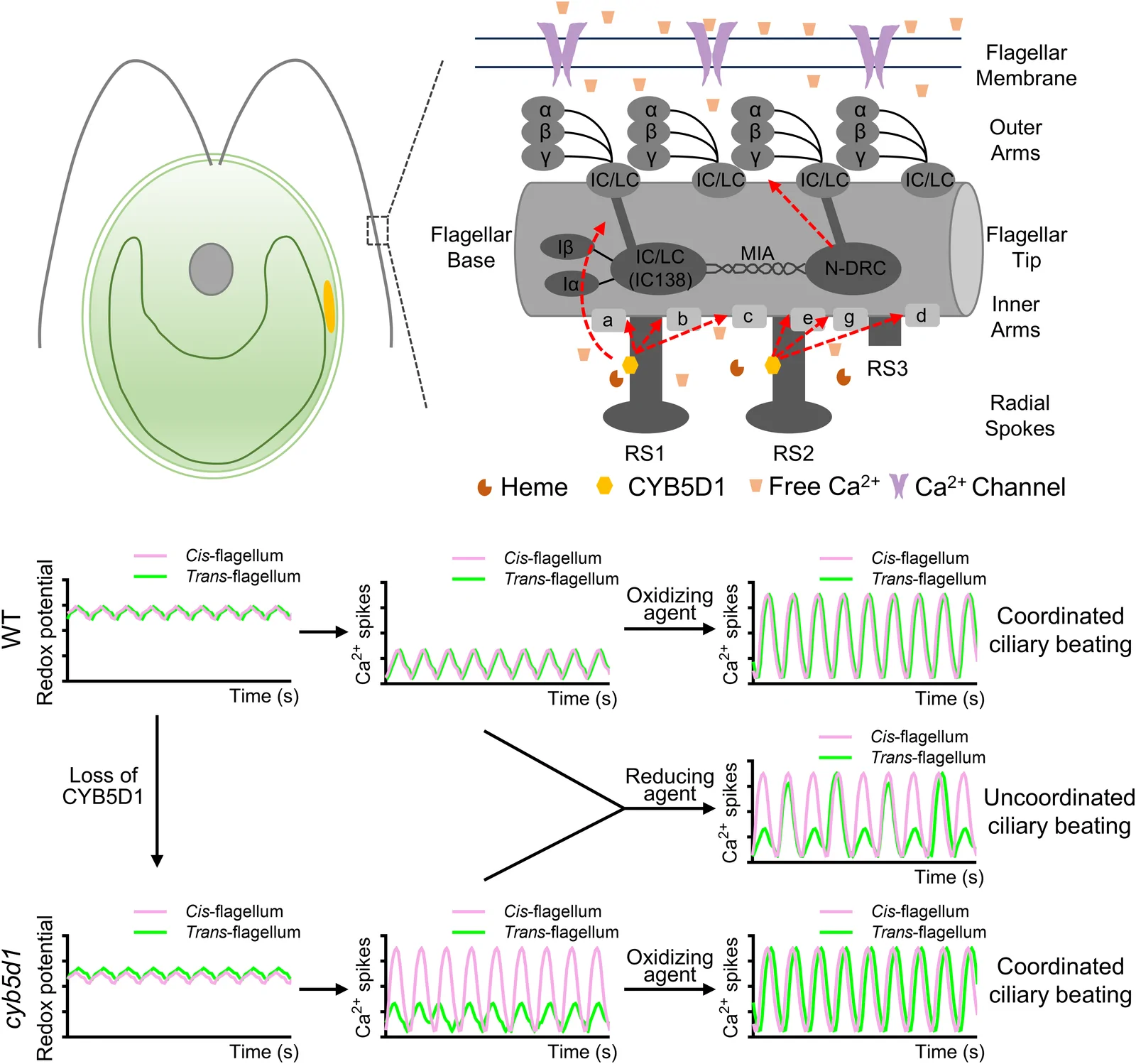
CYB5D1 integrates redox signals to control ciliary coordination through Ca^2+^ dynamics. Model shows hierarchical regulation: 1) CYB5D1 maintains intraflagellar redox homeostasis via heme binding; 2) redox state gates spatiotemporal Ca^2+^ spike patterns; 3) synchronized spikes drive coordinated beating while asymmetric spikes cause discoordination. This mechanism explains how individual cilia achieve synchronized motion and identifies redox as a primary regulatory signal in ciliary biology.

Together with our analyses of swimming and gliding cells, these findings support a hierarchical model in which CYB5D1 couples heme-dependent redox regulation to Ca^2+^ spike dynamics and flagellar coordination (Fig. 7). 1) CYB5D1 functions as a molecular switch that modulates the intraflagellar redox potential, governed by its heme-binding status, and its loss of function induces flagellar hyperreduction. 2) Redox dynamics regulate the Ca^2+^ entering and leaving through channels or pumps in the flagellar membrane, resulting in spatiotemporal Ca^2+^ spiking; hyperreduction elevates *cis*-flagellar spike frequency/amplitude, oxidative stimuli synchronize Ca^2+^ spikes across both flagella, and reductive conditions disrupt synchrony. 3) Ca^2+^ spike geometry dictates mechanical output, as synchronized spikes maintain >84% coordinated beating (IP state), whereas desynchronization reduces coordination of beating to 15% (*SI Appendix*, Fig. S13*C*). Thus, CYB5D1 transduces heme redox signals to calibrate Ca^2+^ spike dynamics, thereby orchestrating metachronal switching between *cis*- and *trans*-flagellar dominance for waveform modification.

## Discussion

In this study, we took advantage of the classical ciliary research model *Chlamydomonas* to dissect the roles of redox and Ca^2+^ in coordinated ciliary beating, and demonstrated that redox kinetics play a critical role in regulating flagellar Ca^2+^ spikes to control coordinated flagellar beating.

### CYB5D1 Maintains the Flagellar Redox State through Binding Heme.

Heme (iron-protoporphyrin IX) is essential for oxidative phosphorylation, oxygen transport, and metabolic regulation (64, 65). Flagellar heme binds the oxidized form of CYB5D1 to maintain the flagellar redox state (21). By stably expressing the redox-sensing protein Kelch::roGFP2-Tsa2ΔC_R_ and anchoring it along the axoneme, we successfully measured the intraflagellar redox potential, and found that deletion of CYB5D1 and disruption of its heme-binding capacity reduce the intraflagellar redox potential (Fig. 3*D*). Consistently, in vitro binding assays demonstrated that CYB5D1 specifically binds hemin which is mediated by the conserved amino acid D58 in CYB5D1 (Fig. 2 *C* and *D*). Therefore, CYB5D1 regulates flagellar redox state through heme binding and release. Furthermore, exogenous reductant (TEMPOL) induced uncoordinated motility and decreased the intraflagellar redox potential in WT, *cyb5d1*, and *cyb5d1* CYB5D1(D58G) mutant cells. Conversely, in the mutant strains, this motility defect was rescued by exogenous oxidant (*t*-BOOH), which caused an increase in the intraflagellar redox potential (Fig. 3*E*). These results demonstrated that exogenous redox agents can alter the flagellar redox potential, which is modulated by CYB5D1.

Accumulating evidence supports the existence of redox signaling pathways within the flagella, which encompass multiple motility-related components. For instance, the central pair subunits PF20 and PP1C (the catalytic subunit C of protein phosphatase type 1) were identified in the thioredoxin interactome (66, 67). Similarly, radial spoke subunits RSP1, RSP9, RSP5, and CYB5D1 also exhibit redox sensitivity (21, 67). Furthermore, the inner dynein arm subunits p28 (IDA4) and IDA5, as well as the α-, β-, and γ-heavy chains of the outer dynein arm, are present in the thioredoxin interactome (67). Moreover, outer dynein arm subunits LC3, LC5, and DC3 are all redox-sensitive proteins (47, 48), implying that their function is modulated by redox changes, and thereby potentially influence the flagellar waveform. Loss of CYB5D1 led to a more reductive state within the flagellum, consistent with reports of a reductive shift in nasal epithelial cells from patients with primary ciliary dyskinesia (PCD) (68). However, this redox imbalance is not a universal consequence of impaired ciliary motility: a radial spoke defective mutant (*pf14*) retained a near WT redox state, whereas outer dynein arm mutants exhibited a similarly reductive shift (*SI Appendix*, Fig. S7). Together, these findings suggest that redox dysregulation is a shared feature of specific ciliary dysfunction subtypes, rather than a general response to motility impairment. The intraflagellar redox state likely reflects the output of a complex regulatory network, determined not solely by the biochemical activity of any single axonemal protein, but by the concerted interactions among multiple redox-active components.

Our measurements of flagellar redox potential in wild-type CC125 (approximately −270 mV) are consistent with those reported by Sugiura et al. (59), who obtained values of approximately −250 mV in the light and −280 mV in the dark using the Oba-Qc probe. The convergence of results across two independent studies using distinct redox sensors validates the reliability of roGFP-based flagellar redox measurements and establishes a robust baseline for interpreting mutant phenotypes. Building on this validated baseline, the D58G point mutant exhibits a complex phenotype distinct from both WT and the *cyb5d1* null mutant, consistent with its classification as a separation-of-function allele. Most strikingly, postspike Ca^2+^ decay kinetics in D58G are markedly slower than in both WT and the null mutant—a phenotype absent in the complete loss of CYB5D1 (Figs. 5*C* and 6 *C* and *F*). We propose that this reflects an “occupancy effect”: the heme-deficient D58G protein, while retaining radial spoke association, physically obstructs downstream components required for Ca^2+^ clearance. In contrast, complete absence of CYB5D1 may activate compensatory redox pathways that sustain near-normal Ca^2+^ extrusion. These findings reveal that CYB5D1 may perform mechanistically separable functions beyond heme binding alone.

### Redox, Ca^2+^, and Ciliary Beating Coordination.

*Chlamydomonas* cells turn toward (positive phototaxis) or move away (negative phototaxis) from the light source by changing flagellar beat frequency and beat pattern (69), which are controlled by redox and Ca^2+^ (21, 29, 40, 42–44). The flagellar beating frequency was reduced when *Chlamydomonas* cells were treated with oxidizing agents (21, 43). Consistently, cells always exhibited positive phototaxis after being treated with oxidizing agents and displayed negative phototaxis after treatment with reducing agents (21, 42, 44). These redox-driven phototactic responses are ultimately mediated through Ca^2+^ signaling. The phototactic response is Ca^2+^-dependent and mediated by signal transduction from the photoreceptor, which is located at the eyespot (70). Activation of the photoreceptor triggers an inward eyespot current and membrane depolarization (70, 71). This event initiates a Ca^2+^ spike that drives an increase in intraflagellar Ca^2+^ and subsequently modulates microtubule sliding between specific doublet pairs, ultimately altering the flagellar waveform (72–75). Notably, studies in sea urchin and starfish sperm demonstrate that increased intraflagellar Ca^2+^ concentration (Ca^2+^ spikes) directly induces directional turns (20). Together, these findings establish that redox and Ca^2+^ play distinct yet coordinated roles in regulating flagellar motility.

In demembranated flagella of *Chlamydomonas*, *cis*–*trans* flagellar dominance is governed by extracellular free Ca^2+^ levels [(21, 29, 30, 40), and this study]. In WT cell models, the *cis*-flagellum beats more strongly than the *trans*-flagellum when Ca^2+^ concentrations are below 10^−8^ M, whereas the *trans*-flagellum beats more strongly than the *cis*-flagellum at 10^−7^ M to 10^−6^ M Ca^2+^ [(21, 29, 30), and this study]. However, in *cyb5d1* cell models, the *trans*-flagellum constantly showed dominance regardless of Ca^2+^ concentration changes [(21), and Fig. 4*A*], a phenotype reminiscent of several non-phototactic *Chlamydomonas* mutants, including *ptx1* (28, 76), *ptx5*, *ptx6*, and *ptx7* (73). Those mutants also lack the normal Ca^2+^-dependent inactivation of the *trans*-flagellum and were postulated to carry axonemal defects. Our previous waveform analysis revealed that the *cyb5d1* mutation primarily distorts the *cis*-flagellum’s recovery stroke, causing it to curl and thereby biasing the cell toward the *trans*-flagellum (21). Thus, lack of the normal Ca^2+^-dependent inactivation of the *trans*-flagellum can result from defects in either the *cis*- or *trans*-flagellum.

Beyond Ca^2+^-dependent regulation, flagellar dominance is also directly modulated by redox status (44). When the redox state switches from a reducing state to an oxidizing state, both WT cells and *agg1* mutants (a negatively phototactic strain) (77) exhibit enhanced *cis*-flagellar dominance—regardless of Ca^2+^ concentration change (44). Remarkably, *cis*-flagellum dominance in models of the mutant *cyb5d1* or *cyb5d1* CYB5D1(D58G) was restored when the reactivation solution was in an oxidizing state, even when Ca^2+^ concentrations were below 10^−8^ M (Fig. 4*B*). This indicates that redox signaling acts upstream of Ca^2+^ in coordinating flagellar beating in *Chlamydomonas*. Consistent with this hierarchy, the oxidizing agent *t*-BOOH induced highly coupled Ca^2+^ spikes between *cis*- and *trans*-flagella in immobilized cells, whereas the reducing agent TEMPOL decreased Ca^2+^ spike coupling (*SI Appendix*, Fig. S11), directly demonstrating that redox kinetics regulate Ca^2+^ spike coordination to control flagellar beating.

The coordination of flagellar beating is ultimately executed through dynein-driven interdoublet microtubule sliding (22–24, 78), and both redox and Ca^2+^ signals converge on axonemal dynein to modulate this process. Redox poise likely affects outer dynein arms—which primarily regulate beat frequency (79)—as evidenced by its differential effects on beat frequency and coordination: oxidizing conditions decrease beat frequency yet enhance interflagellar coordination, whereas reducing conditions leave frequency unchanged but impair coordination (21, 44). Ca^2+^, in turn, may modulate inner dynein arms through centrin-associated subunits and the LC4 light chain of the outer dynein arm, shifting the beat pattern between asymmetric and symmetric waveforms (22, 29, 40, 80). Notably, the docking complex subunit DC3 harbors a vicinal dithiol that enables Ca^2+^ binding exclusively in the reduced state (47), providing a possible molecular node at which redox and Ca^2+^ signals are integrated at the outer dynein arm docking complex. The clinical relevance of this redox–Ca^2+^–dynein axis is underscored by the finding that loss of the thioredoxin-domain proteins TXNDC3 and TXNDC6 causes PCD in humans (50). Together with our findings on CYB5D1, this suggests that redox dysregulation may represent a recurrent, conserved mechanism underlying ciliary motility disorders.

Due to the limited spatiotemporal resolution of our imaging system, we were unable to quantify flagellar Ca^2+^ spikes in beating flagella. Previous work in sea urchin sperm has established that a Ca^2+^ spike directly triggers a turning maneuver by altering the flagellar waveform (20), confirming the direct role of Ca^2+^ in steering. A recent study further reveals that the *cis*-flagellum acts as the dominant regulator that coordinates the beating pattern, while the *trans*-flagellum largely follows its motion in *Chlamydomonas* (81). This regulatory hierarchy likely explains why the loss of CYB5D1 primarily manifests as motility defects in the *cis*-flagellum (21). In summary, we have established CYB5D1 as a critical integrator of heme-mediated redox dynamics and Ca^2+^ signaling in the flagella of *Chlamydomonas*. Our data support a model in which a redox-Ca^2+^-based regulatory system modulates flagellar beat waveforms through coordinated changes in redox state and Ca^2+^-dependent signaling.

## Materials and Methods

The full procedures for algal cell culture and strains; gene cloning and transformation; flagella isolation and fractionation; live-cell imaging; quantifying axonemal Kelch::MatryoshCaMP6s; GCaMP6s intensity analysis; swimming track recording and assessment of swimming velocity; analysis of flagellar swimming behavior and beat frequency; SDS-PAGE and immunoblotting; induced expression and purification of GST::CYB5D1; establishment of a redox standard curve based on fluorescence intensity; UV absorption spectroscopy and SDS-PAGE in-gel redox assay; cellular demembranation and reactivation are provided in *SI Appendix*. All data generated in this study are included in the article and/or supporting information.

## Supplementary Material

Appendix 01 (PDF)

Movie S1. Confocal video showing the flagellar Ca^2+^ dynamics of CC125, *cyb5d1* and *cyb5d1* CYB5D1(D58G).

Movie S2. Confocal video showing the flagellar Ca^2+^ dynamics of CC125, *cyb5d1* and *cyb5d1* CYB5D1(D58G) under 1 mM EGTA treatment.

Movie S3. Confocal video showing the flagellar Ca^2+^ dynamics of CC125, *cyb5d1* and *cyb5d1* CYB5D1(D58G) under 0.4 mM oxidant *t*-BOOH treatment.

Movie S4. Confocal video showing the flagellar Ca^2+^ dynamics of CC125, *cyb5d1* and *cyb5d1* CYB5D1(D58G) under 40 mM reductant TEMPOL treatment.

Movie S5. Confocal video showing the flagellar Ca^2+^ dynamics of CC125, *cyb5d1* and *cyb5d1* CYB5D1(D58G) under treatment with 0.4 mM oxidant *t*-BOOH in the presence of 1 mM EGTA.

Movie S6. Confocal video showing the flagellar Ca^2+^ dynamics of CC125, *cyb5d1* and *cyb5d1* CYB5D1(D58G) under treatment with 40 mM reductant TEMPOL in the presence of 1 mM EGTA.

Movie S7. HS211 flagellar beat frequency example.

## Data Availability

Study data are included in the article and/or supporting information.
