# Supplementary material for: Heme-binding protein CYB5D1 couples intraflagellar redox to calcium signaling for coordinated flagellar beating
Source: Proc Natl Acad Sci U S A. 2026 Aug 19;123(34):e2535939123. doi: 10.1073/pnas.2535939123 (PMC13505981; doi:10.1073/pnas.2535939123)
Supplement: Supplementary file 1 [file pnas.2535939123.sapp.pdf]

PNAS

Supporting Information for
Heme-binding Protein CYB5D1 Couples Intraflagellar Redox to Calcium Signaling for Coordinated Flagellar Beating.

Yiwen Lin[a,b], Lijuan Zhao[c], Gai Liu[a], Xuan Deng[a], Stephen M. King[d], and Kaiyao Huang[a].

Corresponding author: Stephen M. King and Kaiyao Huang.

Email: king@uchc.edu, huangky@ihb.ac.cn

This PDF file includes:

Supporting Materials and Methods
Figures S1 to S14
Tables S1
Legends for Movies S1 to S7
SI References

Other supporting materials for this manuscript include the following:

Movies S1 to S7

Materials and Methods

Cell Culture and Strains.

Chlamydomonas reinhardtii wild-type strain (CC125), HS450 (mCh::CYB5D1 *cyb5d1*), HS447 (*cyb5d1*) (1) and other transgenic strains were grown in liquid Tris-acetate-phosphate (TAP) (2) or on solid TAP agar (1.5%) plates under continuous illumination (30 $\mu E \cdot m^{-2} \cdot s^{-1}$) at 22°C. Transformants were selected on TAP agar (1.5%) medium containing 2.5 $\mu g \cdot mL^{-1}$ zeocin or 25 $\mu g \cdot mL^{-1}$ hygromycin. To ensure that the algae strains have the same genetic background, we crossed the CC124 (mt-) strain with *cyb5d1* (CYB5D1 D58G) (mt+) to replace IFT46::YFP with IFT46, then the progeny was used as chassis cells to express redox and Ca^{2+} sensors.

Gene cloning and transformation.

The primers for amplifying the CYB5D1ΔN and CYB5D1ΔC genes were: CY-N-F 5′-CGCGATATCCACTGGTTCGACCCCACCAC-3′, CY-N-R 5′-CGCGAATTCGGCCACGGTGAGG TCGTCGT-3′, and CY-C-F 5′-CGCGATATCATGGCGCCTCCGCGTGGG-3′, CY-C-R 5′-CG CGAATTCCTTCCAAGTGTAGCTCTTGGCGT-3′ respectively. The primers used to amplify the D58G point mutation of *CYB5D1* were: D58G-F 5′-AGCGGCTGGGCAGGGCCTCACACACTGGT-3′, and D58G-R 5′-ACCAGTGTGTGAGGCCCTGCCCAGCCGCT-3′. The primer Kelch-F and Kelch-R used to amplify the flagellar anchoring protein were 5′-AACATCTTAAACATATGGTGC GCTTGGAGCAACG-3′, and 5′- CGCTGGCCATGAATTCCGGGCCGATGATGGGGG-3′.

In order to express truncated CYB5D1, the CYB5D1ΔN and CYB5D1ΔC regions were amplified from the CC125 cDNA with the restriction enzyme sites *Eco*RV and *Eco*RI added. After digestion, the CYB5D1ΔN or CYB5D1ΔC fragment was inserted behind mCherry in plasmid pHK486 (1), which contains the *HSP70-RBCS2* fusion promoter, the selectable marker gene *ble*, and the 3′ UTR of *RBCS2*, thus yielding the plasmids pHK942 and pHK945. The pHK942 and pHK945 constructs were linearized with *Sca*I and transformed into *cyb5d1* mutant by electroporation. To express the D58G point mutant form of *CYB5D1*, *CYB5D1* was amplified from CC125 genomic DNA with the restriction enzyme sites *Eco*RV and *Eco*RI added, then ligated to the pMD-18T vector. Next, the point mutations in *CYB5D1-T* were amplified and digested with *Dpn*I to remove template DNA and then transferred to *E. coli* to replicate the point mutation plasmid of *CYB5D1-T*. Finally, the truncated *CYB5D1* in pHK942 was replaced by the point mutation form of *CYB5D1*, thus yielding the plasmid pHK971 (D58G).

To express the exogenous redox and Ca^{2+} sensors within the flagella, the two sensor sequences were reverse-translated using the most highly used *Chlamydomonas* codons (3, 4), and then three *Chlamydomonas RBCS2* introns were inserted (*roGFP2-Tsa2ΔC*$_R$: CAA-intron 1-GAT, ACG-intron 2-GCA, and GGC-intron 3-CTG, *MatryoshCaMP6s*: AAG-intron 1-GTG, GAG-intron 2-GAC, and AGG-intron 3-GCA) as previously suggested for foreign genes (5, 6). Next, the redox and Ca^{2+} sensors were fused to the C-terminus of the Kelch repeat domain, which is a part of α-HC (heavy chain) (the N-terminal 333aa). The plasmids also contain the *HSP70-RBCS2* fusion promoter, the selectable marker gene *APHVII*, *Multi-Stop* sequence and the 3′ UTR of *RPL23*, thus yielding the plasmid pHK984 and pHK985. Then, pHK984 or pHK985 plasmids were linearized with *Not*I and transformed into CC125, *cyb5d1*, *cyb5d1*(CYB5D1 D58G) strains.

Flagella isolation and fractionation.

100 mL *Chlamydomonas* cells were grown in constant light to a density of 4-5×10^6 cells/mL in TAP medium, then flagella were isolated by the pH shock method (7) and resuspended in HMDEK buffer (20 mM HEPES pH 7.2, 5 mM $MgCl_2$, 1 mM EDTA, 1 mM DTT containing complete protease inhibitor cocktail from Roche). For flagellar fractionation, isolated flagella were incubated with 0.5% NP-40 (Calbiochem) for 30 minutes at room temperature (25°C) to remove the membrane plus matrix, and then centrifuged at 10,000 g for 10 minutes at 4°C to yield flagellar axonemes. The pelleted axonemal fraction was resuspended in the same volume of HMDEK buffer for western blots.

Identification of *cis*- and *trans*-flagella in *Chlamydomonas* cells

To distinguish the two flagella of *Chlamydomonas*, the eyespot was first identified using the bright-field channel of a Leica TCS SP8 STED confocal microscope. The flagellum located closer to the eyespot was identified as the *cis*-flagellum, whereas the flagellum farther from the eyespot was identified as the *trans*-flagellum (8). This assignment was performed for each cell immediately prior to imaging or analysis to ensure accurate classification.

Live-cell imaging and Ca^{2+} spike frequency analysis.

All live-cell imaging was acquired using a Leica TCS SP8 STED confocal microscope equipped with a 100× oil objective (Leica Microsystems, Germany) under static conditions. The imaging of freely swimming algal cells was acquired using a Nikon AXR confocal microscope equipped with a 100× oil objective (Nikon ECLIPSE Ti-U, Nikon, Japan). Fluorescent signals of mCherry (excitation/emission 587 nm/600-650 nm), YFP (excitation/emission 514 nm/515-560 nm) and roGFP2 (excitation/emission 405 nm and 488 nm/500-540 nm), EGFP (excitation/emission 488 nm/500-540 nm) and LSSmOrange (excitation/emission 458 nm/600-650 nm) were processed using Leica software LAS X or Fiji. To better observe the redox potential within the flagella of *Chlamydomonas*, a slide preparation was first made: double-sided tape with a width of 3 mm and a thickness of 150 μm was customized. The protective layer was peeled off, and the tape was attached to the edges of a 24 × 24 mm coverslip. This side was then adhered to a clean microscope slide. 50 μL of algal cells in the logarithmic growth phase were carefully pipetted along the edge, and the cells were drawn into the chamber by capillary action. Taking advantage of the glycoprotein-rich nature of *Chlamydomonas* flagella, the flagella of the algal cells can adhere to the glass surface without requiring any chemical modification. To assess the effects of redox reagents on intraflagellar redox potential in *Chlamydomonas*, algal cells were treated with 0.4 mM *t*-BOOH (tert-butyl hydroperoxide) or 40 mM TEMPOL (4-hydroxy-2,2,6,6-tetramethylpiperidine 1-oxyl) for 1 minute and subsequently imaged by STED confocal microscope.

To observe the Ca^{2+} spikes within the flagella of *Chlamydomonas* cells under static conditions, 1% agarose was used to immobilize the algal cells. The specific method is as follows: 0.1 g of agarose was dissolved in 10 mL of distilled water, heated in a microwave until the agarose was completely dissolved, and then kept warm at 60°C in a metal bath for later use. A 3 mL Pasteur pipette was used to aspirate an appropriate amount of agarose, which was then dropped onto a 24 × 50 mm coverslip (forming a liquid surface with a diameter of about 1 cm). After waiting for 5-10 seconds (the exact time can be adjusted based on ambient temperature), another 24 × 50 mm coverslip was quickly placed on top in a crosswise manner. After 30 seconds, the top coverslip was removed, and 3-4 μL of the algal cell sample was added to the center of the agarose. The coverslip was then placed back on top, resulting in a standard microscopy slide for observation.

For Ca^{2+} spike analysis, time-lapse fluorescence images of flagella expressing MatryoshCaMP6s were acquired at 5.22 frames per second under static conditions. For each experiment, regions of interest (ROIs) corresponding to the *cis*- and *trans*-flagellum were manually selected, and the mean fluorescence intensity over time was measured using Fiji (ImageJ). A Ca^{2+} spike was defined as a transient elevation in fluorescence intensity exceeding three times the intensity of the baseline. Baseline intensity was calculated as the median fluorescence over a moving window (30 time points, ~5.7 s) excluding peak events, as described previously (9). Spike frequency was then calculated as the number of such events per minute for each flagellum. All analyses were performed blind to genotype.

In order to observe the influx of calcium ions within the flagella of *Chlamydomonas* cells in a freely swimming state, the *Chlamydomonas* cells were resuspended in a solution containing 0.05% 10 μm silica beads and a final concentration of 10% Ficoll-400. This was done to reduce the flagellar beat frequency (to approximately 20 Hz) and to confine the swimming space of the *Chlamydomonas* cells (thereby increasing the probability of the cells appearing in the focal plane of the microscope). The microscope region of interest (ROI) was set to 256×56, with a capture speed of 256 fps.

Quantifying axonemal Kelch::MatryoshCaMP6s.

To quantify the GCaMP6s reactivity, axonemes were isolated from the *cyb5d1* (Kelch::MatryoshCaMP6s) or CC125 (Kelch::MatryoshCaMP6s) strains. Flagellar isolation and demembranation were performed as mentioned above. For Ca^{2+} titrations, GCaMP6s axonemes were washed and resuspended in 30 mM MOPS, pH 7.2, 100 mM KCl with either 10 mM EGTA (zero free Ca^{2+}) or 10 mM CaEGTA (39 µM free Ca^{2+}). The recipe of 10 mM CaEGTA was adopted from Cold Spring Harbor Protocols (10) and then observed on the STED confocal microscope (Leica Microsystems, Germany). GCaMP6s intensity was measured using Fiji software, following a published protocol (11). Each axoneme was traced by hand using the Segmented line tool with a 7-pixel line width, analyzed by the Plot Profile function, and then the average intensity per pixel was calculated. At least 15 axonemes were analyzed for each calcium concentration.

GCaMP6s intensity analysis.

For quantitative analysis of GCaMP6s fluorescence intensity, flagellar structures were manually segmented using a region-of-interest (ROI) tracing protocol in Fiji/ImageJ (v2.3.0). Time-lapse imaging data of Kelch::MatryoshCaMP6s-expressing samples were processed to generate kymograms through the Kymograph Builder plugin, enabling spatiotemporal visualization of calcium dynamics along the flagellar axis. Spatial calibration was performed using a 0.5 µm/pixel reference grid, and intensity profiles were normalized to background fluorescence measured in adjacent non-flagellar regions.

Swimming track recording and assessment of swimming velocity.

To facilitate high-resolution observation of freely swimming *Chlamydomonas* cells, the algal suspension was loaded into a 150-µm-deep microfluidic chamber (described above). Bright-field microscopy with a 20× objective lens was employed to record the motility patterns, with cellular movements captured with a digital camera incorporating a charge-coupled device (Digital Sight DS-U3). Three-dimensional trajectory reconstruction was performed through Z-stack image processing in Fiji/ImageJ (v2.3.0), followed by quantitative swimming velocity analysis using the TrackMate plugin under controlled hydrodynamic conditions.

Analysis of flagellar swimming behavior and beat frequency.

To systematically analyze flagellar beat patterns and flagellar beat frequencies in *Chlamydomonas reinhardtii*, algal suspensions were introduced into a 150-µm-depth microfluidic chamber (previously optimized for protist motility studies). To eliminate photophobic responses induced by white light exposure, a 620-nm long-pass optical filter was integrated into the bright-field illumination path. High-speed videography (Motion-BLITZ EoSens mini1; Mikrotron, Germany, 500 fps) was performed using a 100× oil-immersion objective (NA 1.4) to capture flagellar dynamics at sub-millisecond resolution. Flagellar waveforms were categorized into three distinct modes based on established criteria: IP, AP or Slip (1). To determine the beat frequency, cells with exactly 15 continuous beat cycles were selected, and the mean beat frequency was calculated by dividing the elapsed time by 15 for each cell. At least 50 cells were measured. The statistical significance was determined using Student′s *t*-test ($\alpha = 0.05$).

SDS-PAGE and immunoblotting.

The procedures for SDS-PAGE and immunoblotting were as described previously (2). Antibodies used were: mouse mCherry (1:2,000, Earthox), mouse anti-α-tubulin (1:50,000, Sigma), rabbit RSP1 (1:2,000), mouse GFP (1:500), rabbit IFT46 (1:2,500). The IFT46 antibody was custom-generated in our laboratory.

Induced expression and purification of GST::CYB5D1.

The *CYB5D1* coding sequence was synthesized and subsequently cloned into pGEX-2T vector (Sangon Biotech, Shanghai, China). The recombinant plasmid was transformed into *E. coli* BL21(DE3) competent cells for protein expression. Bacterial cultures were grown in LB medium at 37°C until OD_{600} reached 0.6–0.8. Protein expression was induced by adding 0.3 mM IPTG, followed by incubation at 25°C for 16-18 hours to enhance soluble protein yield. Pelleted cells were resuspended in ice-cold PBS (pH 7.4) containing 1% Triton X-100 and 1 mM PMSF. Ultrasonication was performed on ice (3-s pulse/6-s interval for 10 min) to lyse cells. Lysates were centrifuged at 12,000g for 30 min at 4°C to remove debris. The supernatant was mixed with pre-equilibrated glutathione magnetic beads, and protein purification was performed following the protocol provided with the Mag-MK GST Fusion Protein Purification Kit (Sangon Biotech, Shanghai, China, No.C650013). Purity was assessed by SDS-PAGE and Coomassie blue staining.

Establishment of a redox standard curve based on fluorescence intensity.

Flagella isolation and demembranation were performed following the methodology previously described in our experimental protocol. Isolated flagella were resuspended in the redox buffers, containing different ratios of oxidized- and reduced-dithiothreitol (denoted DTT_{ox} and DTT_{red}, respectively), 30 mM Hepes-NaOH (pH 7.4), 5 mM MgSO4, 50 mM Potassium acetate and 1% polyethylene glycol (Mw: 20,000) (Table S1) (12). The redox potential of each buffer was calculated from the Nernst equation. Fully oxidized or reduced control solutions, containing 1 mM diamide or 10 mM DTT respectively, were prepared as experimental controls. To minimize liquid flow effects during flagellar observation, 3-4 μL flagellar samples were immobilized on 1% agarose pads following the established protocol, followed by imaging using a STED confocal microscopy system. For live-cell fluorescence intensity analysis within flagella, cells in 150 μm-deep observation chambers were imaged by STED. The glycoprotein-rich nature of flagella facilitated passive adsorption onto glass substrates, followed by super-resolution imaging conducted via STED confocal microscopy. RoGFP2 exhibits two excitation maxima at 405 nm and 488 nm with fluorescence emission monitored at 510 nm (500-540 nm). The “blank corrected” values were taken to calculate the degree of sensor oxidation (OxD) using the following equation (6, 13):

$$\text{OxDroGFP2} = \frac{I405_{sample} \times I488_{red} - I405_{red} \times I488_{sample}}{I405_{sample} \times I488_{red} - I405_{sample} \times I488_{ox} + I405_{ox} \times I488_{sample} - I405_{red} \times I488_{sample}}$$

where “I” is the fluorescence intensity at 510 nm after excitation at either 405 nm or 488 nm. The subscripts “ox”, “red”, and “sample” indicate the fluorescence intensity measured for the fully oxidized and fully reduced controls and the sample, respectively.

Hemin binding assay.

The binding of CYB5D1 with hemin was monitored by UV absorption spectroscopy and heme-catalyzed peroxidase reaction using a previously published protocol (14). A 1 mM hemin solution was prepared by dissolving 0.0013 g hemin (Sigma-Aldrich) in 200 μL of 0.1 M NaOH in a 2 mL microcentrifuge tube, followed by dilution with 1.8 mL PBS (pH 7.4). For each reaction, 5 μg of purified CYB5D1-related protein or BSA was incubated with 5 μL of the 1 mM hemin solution (final hemin concentration 5 nM) at room temperature for 30 min.

For UV absorption spectroscopy, an enzyme microplate reader was set to “Spectra” scanning mode with a range of 300–700 nm and a 1 nm interval. Background scans of hemin and protein solutions were recorded, followed by scans of the mixed solution.

For the SDS-PAGE in-gel redox assay, the sample was mixed with a non-reducing protein loading buffer (without β-mercaptoethanol) and subjected to electrophoresis at constant voltage. The resolving gel was then rinsed with PBS (pH 7.4) for 5 min. An ECL substrate was prepared by mixing two chromogenic solutions in a 1:1 ratio (typically 1 mL each per gel). The mixed substrate was applied evenly onto the gel surface and incubated for 1–2 min. Finally, the gel was sealed with plastic wrap and exposed to X-ray film for autoradiography.

Cellular demembranation and reactivation.

To analyze flagellar responsiveness under Ca^{2+} regulation, cells were demembranated and reactivated with protocol modifications as follows (8). Briefly, a 15 mL cell suspension (1×10^7 cells/mL) was centrifuged at 500g for 3 min. The pellet was washed three times with membrane stabilization buffer (10 mM HEPES pH 7.4, 0.5 mM EGTA) and treated with ice-cold demembranation solution (5×pellet volume containing: 30 mM HEPES pH 7.4, 5 mM $MgSO_4$, 1 mM DTT, 1 mM EGTA, 25 mM KCl, 0.5% PEG 20,000, 1% Ficoll 400, 0.1% NP-40) for 5 min. Gentle resuspension was performed using trimmed pipette tips to preserve axonemal integrity, and demembranated cells were utilized within 3 h. For reactivation, 100 μL demembranated cells were centrifuged at 1,500g for 30 s and resuspended in 300 μL standard reactivation buffer (30 mM HEPES pH 7.3, 5 mM $MgSO_4$, 1 mM DTT, 25 mM KCl, 2% PEG 20,000, 2 mM EGTA, 1 mM ATP). To investigate the effects of redox agents on flagellar reactivation, reactivation solutions containing either 0.4 mM oxidizing agent *tert*-butyl hydroperoxide (*t*-BOOH) or 40 mM reducing agent TEMPOL (4-hydroxy-TEMPO) were prepared. Free Ca^{2+} concentrations were precisely adjusted using a Ca-EGTA buffering system. Motility analysis was conducted via phase-contrast microscopy (Olympus U-HGLGPS) with quantitative assessment of rotational polarity as previously validated.

Fig. S1

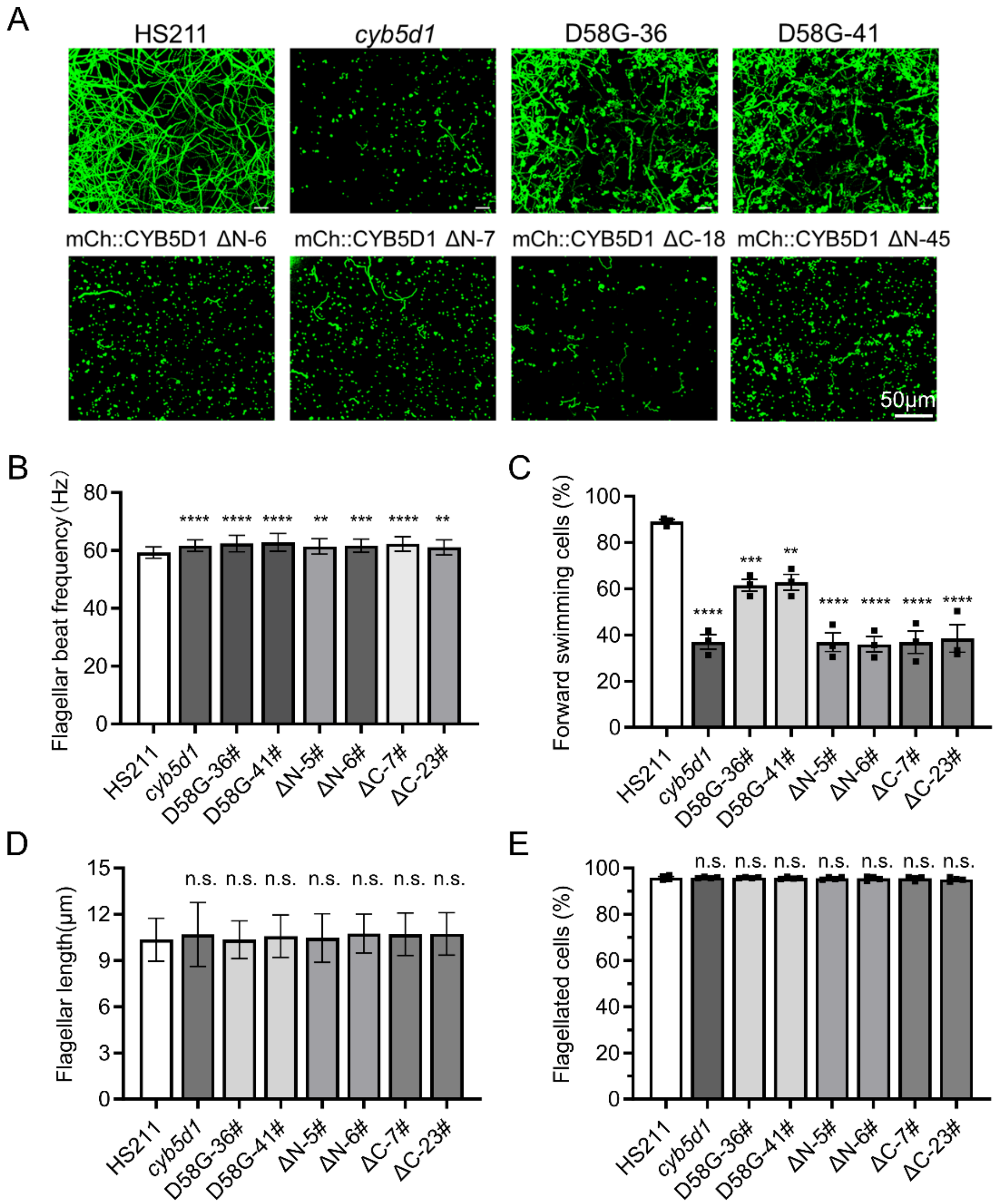

Fig. S1. Motility analysis of CYB5D1 mutants. (*A*) The movement trajectories of *Chlamydomonas* cells expressing the point mutated and truncated CYB5D1 proteins. Scale bar: 50 μm. (*B*) Mean flagellar beat frequency in the indicated strains. (*C*) Mean forward swimming speeds in the indicated strains. (*D*) Mean flagellar lengths in the indicated strains. (*E*) Mean flagellated cells in the indicated strains. Data are presented as mean ± SD (error bars). one-way ANOVA with Tukey's test compared to wild-type. n.s. indicates no significant difference. ****$p <$ 0.0001. HS211 is the parental strain of *cyb5d1* mutant.

Fig. S2

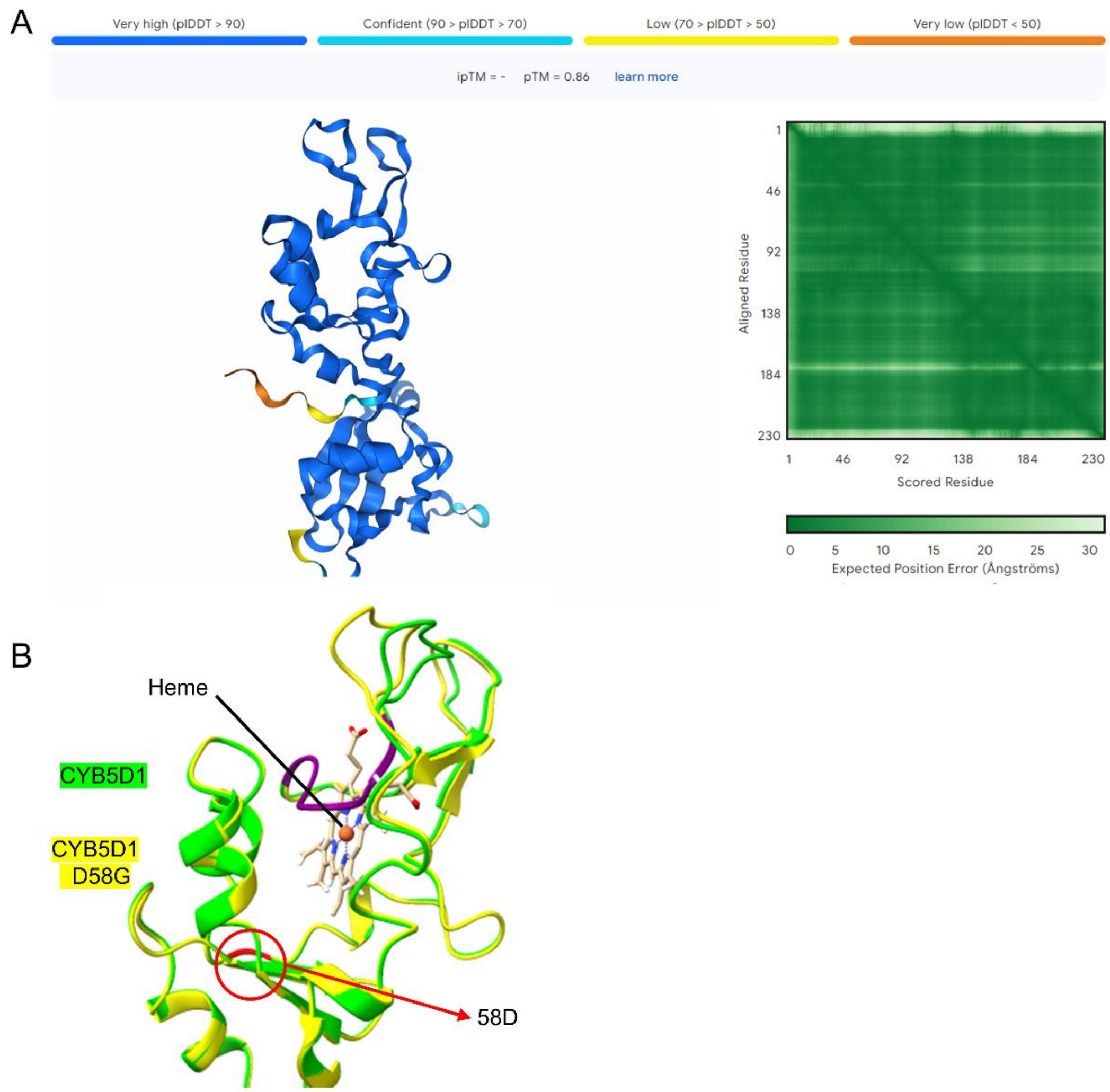

Fig. S2. Comparison of the structure of mutated CYB5D1(D58G) and CYB5D1. (*A*) AlphaFold-predicted structure of CYB5D1(D58G). (*B*) Structural overlay: Published CYB5D1 (PDB **7JTK**, green) vs. D58G mutant (yellow). The heme molecule (red sticks) is modeled into the binding pocket. Residues 89–95 (purple) form a loop that occludes the pocket in the absence of heme. The mutation site (D58) is highlighted. Structural modeling of the D58G mutant revealed that the conformation of the 89–95 loop remains largely unchanged; however, the substitution leads to a marked constriction of the heme binding pocket.

Fig. S3

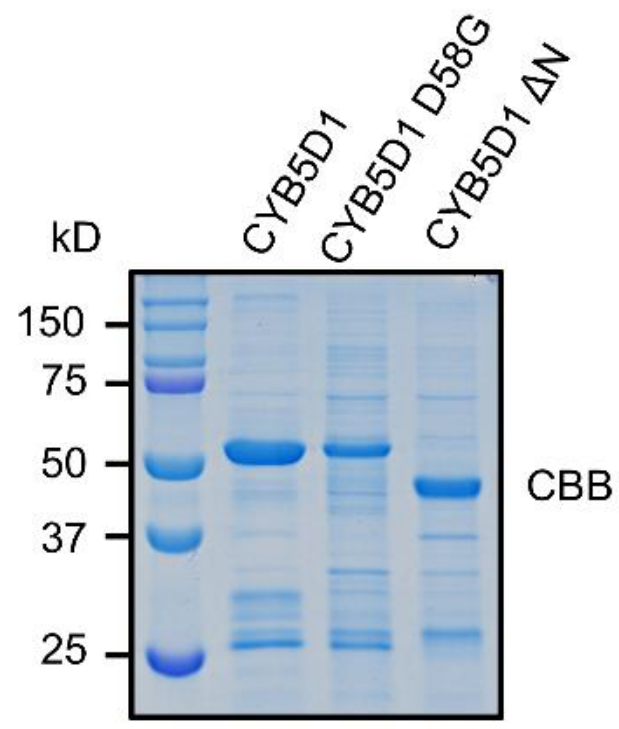

Fig. S3. Expression and purification of CYB5D1 variants. Purified GST::CYB5D1 and mutated GST::CYB5D1 proteins (5 μg each) stained with Coomassie blue staining. Molecular weight markers are at left.

Fig. S4

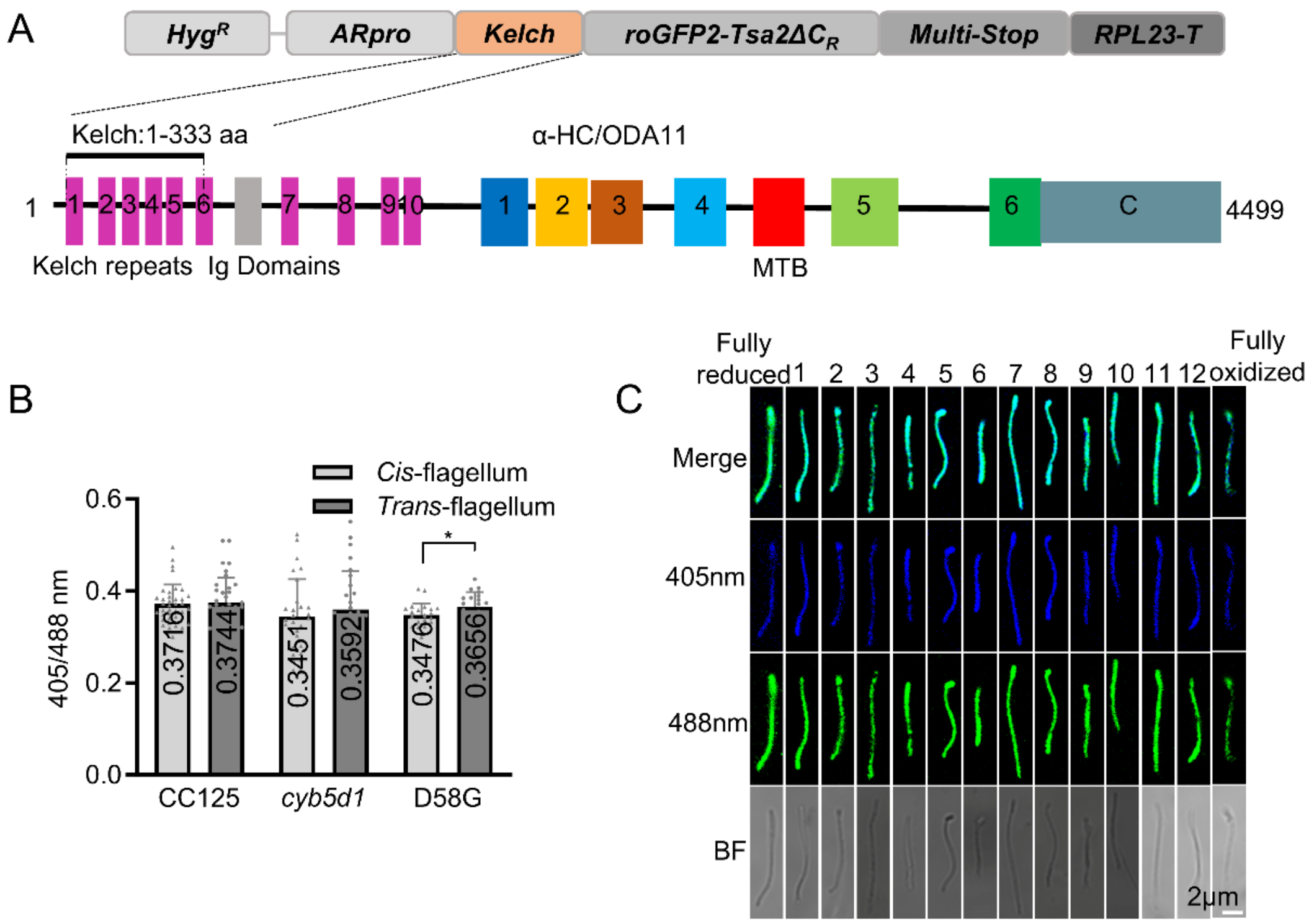

Fig. S4. Flagellar redox symmetry and sensor calibration. (*A*) Schematic diagram of the flagellar redox-sensing construct. (*B*) The fluorescence intensity of *cis*- and *trans*-flagella in the indicated strains was statistically analyzed. Statistical analysis was performed using two-way ANOVA with Šidák's post-hoc test. Data are presented as mean ± SD. N > 21 per strain. (*C*) Isolation and fluorescence imaging of flagella from *Chlamydomonas* cells expressing Kelch::roGFP2-Tsa2ΔC$_R$. Isolated flagella were treated with 0.2% NP-40 for demembranation, followed by fluorescence imaging in the presence of 10 mM DTT (fully reduced), 1 mM Diamide (fully oxidized), or a series of redox buffers (refer to Table S1).

Fig. S5

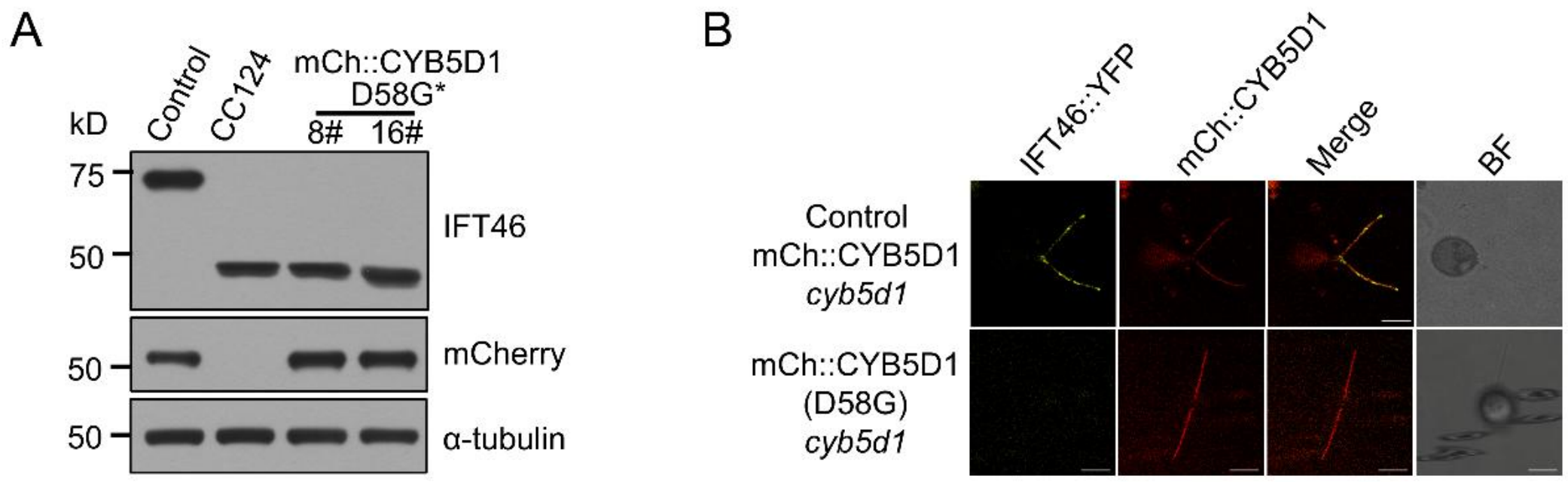

Fig. S5. Validation of IFT46 replacement and CYB5D1 localization in mutant strains. (*A*) Western blot was used to check if IFT46::YFP was replaced by IFT46 in mCh::CYB5D1 D58G (*cyb5d1*) strain by crossing to CC124. The top panel uses an anti-IFT46 antibody to detect IFT46 expression in whole-cell lysates, confirming successful replacement of IFT46::YFP with IFT46 in the point-mutant strains. The middle panel, using an anti-mCherry antibody, confirms that cells maintain the mutated CYB5D1. The bottom panel shows α-tubulin as a loading control. (*B*) Fluorescence imaging of flagella and cells expressing mCh::CYB5D1 and mCh::CYB5D1 D58G (*cyb5d1*). The results indicate that we successfully replaced IFT46::YFP with untagged IFT46 in the mCh::CYB5D1 D58G (*cyb5d1*) strain. Scale bar: 5 µm.

Fig. S6

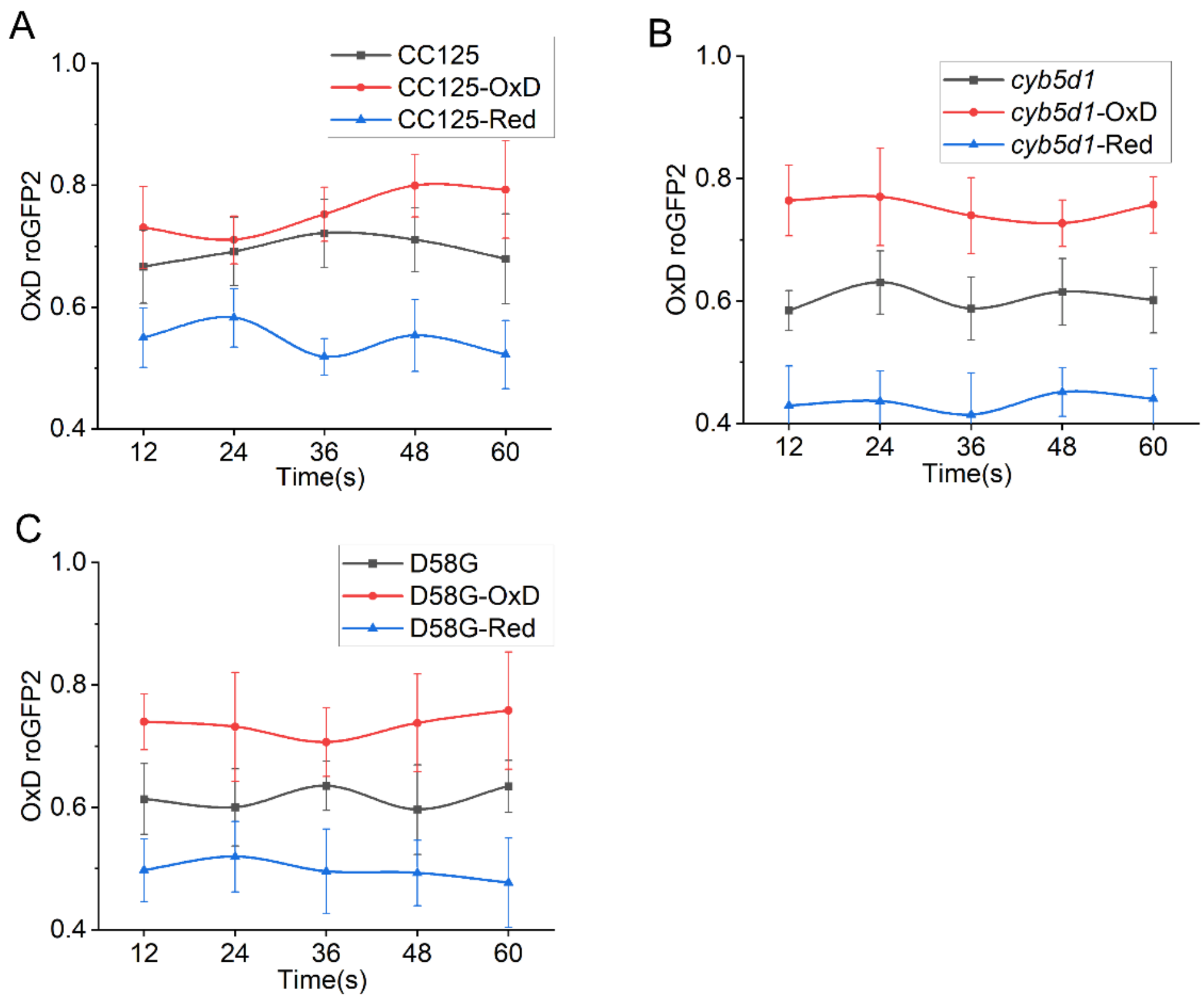

Fig. S6 Transient redox responses in wild-type and *cyb5d1* mutants. Summary of the redox changes of live cells expressing Kelch::roGFP2-Tsa2ΔCR within 1 min under indicated conditions: untreated (Control), 0.4 mM *t*-BOOH (oxidant), or 40 mM TEMPOL (reductant). Three strains are shown: (*A*) Wild type (CC125), (*B*) *cyb5d1* mutant, (*C*) *cyb5d1* (mCh::CYB5D1 D58G) mutant. Data are presented as mean ± SD. Statistical analysis was performed using two-way ANOVA with Tukey's post-hoc test to assess the effects of strain and treatment. N > 10 per condition.

Fig. S7

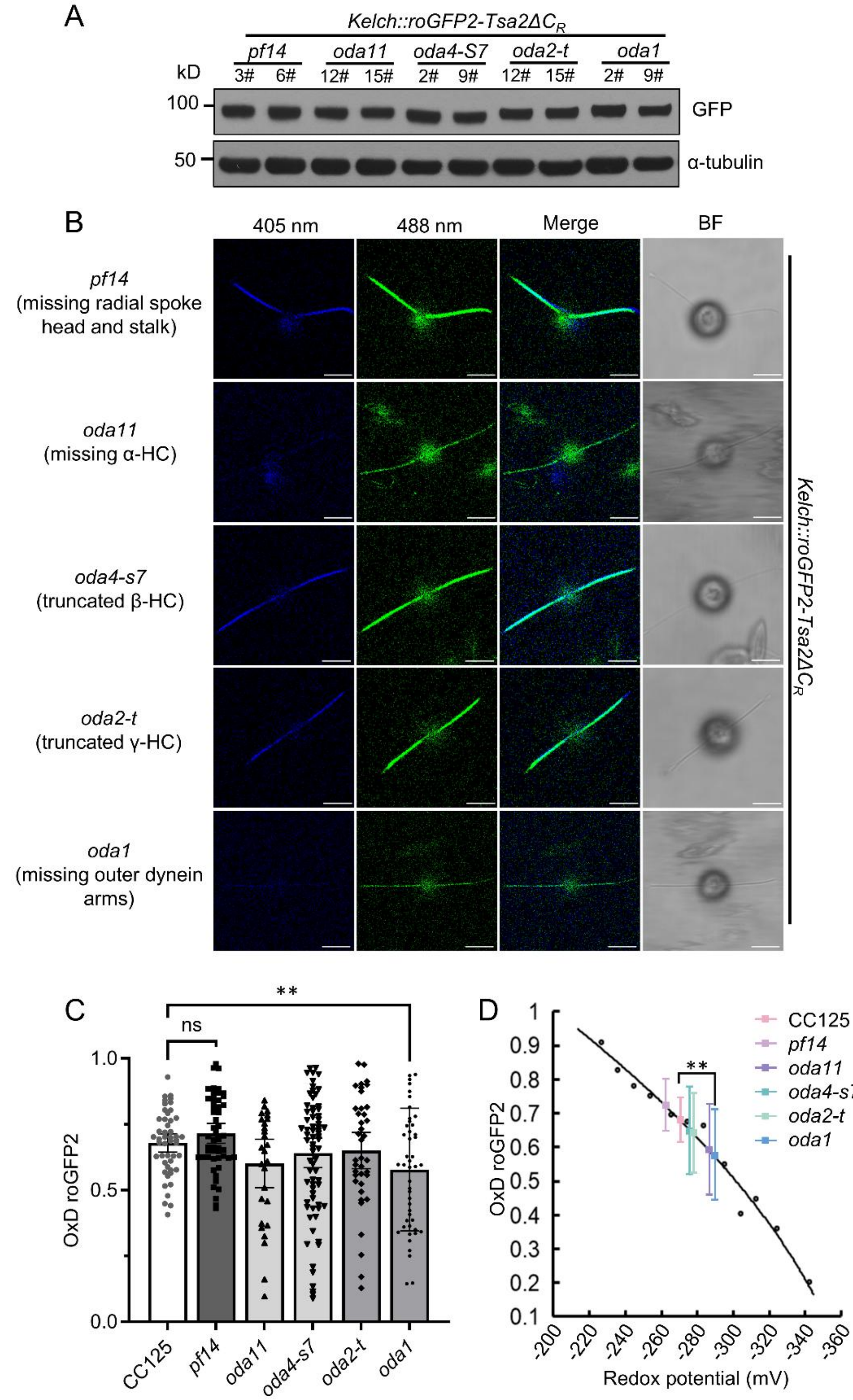

Fig. S7 Flagellar redox potential in motility defective *Chlamydomonas* mutants. (*A*) The expression of Kelch::roGFP2-Tsa2ΔC$_R$ in *pf14*, *oda11*, *oda4-s7*, *oda2-t* and *oda1* mutants. The fusion protein was detected using an anti-GFP antibody and α-Tubulin served as a loading

control. (*B*) Representative live-cell fluorescence imaging of Kelch::roGFP2-Tsa2ΔC_R in the indicated strains. Note that *oda11* (lacking α-HC) and *oda1* (missing outer dynein arms) showed reduced flagellar signal, likely due to impaired anchoring of the Kelch domain, which is derived from the N-terminus of α-HC. Since we used ratiometric measurements, this signal reduction has only a minor impact on the results. Scale bar: 5 μm. (*C*) Quantitative analysis of the roGFP2 oxidation ratio (OxD) in the flagella of *pf14, oda11*, *oda4-s7 and oda1* mutants. Compared to wild-type CC125, *pf14* (radial spoke-defective) showed a slight increase in OxD (more oxidized), but this increase was not statistically supported ($p > 0.05$). In contrast, all outer dynein arm mutants (*oda11*, *oda4-s7*, *oda2-t*, and *oda1*) exhibited reduced OxD values (more reduced), with *oda1* showing the most pronounced effect. Data are presented as mean ± SD; n > 30 cells per strain; one-way ANOVA with Tukey's test compared to wild-type; Statistical significance was assessed at $\alpha = 0.05$; $^{**}p < 0.01$; ns, not significant. (*D*) Calculated flagellar redox potentials derived from the calibration curve established in Fig. 3D. The values were: wild-type CC125, −269.56 ± 12.53 mV; *pf14*, −263.45 ± 14.08 mV; *oda11*, −284.52 ± 22.72 mV; *oda4-s7*, −277.45 ± 24.06 mV; *oda2-t*, −275.69 ± 21.05 mV; *oda1*, −288.51 ± 21.96 mV.

Fig. S8

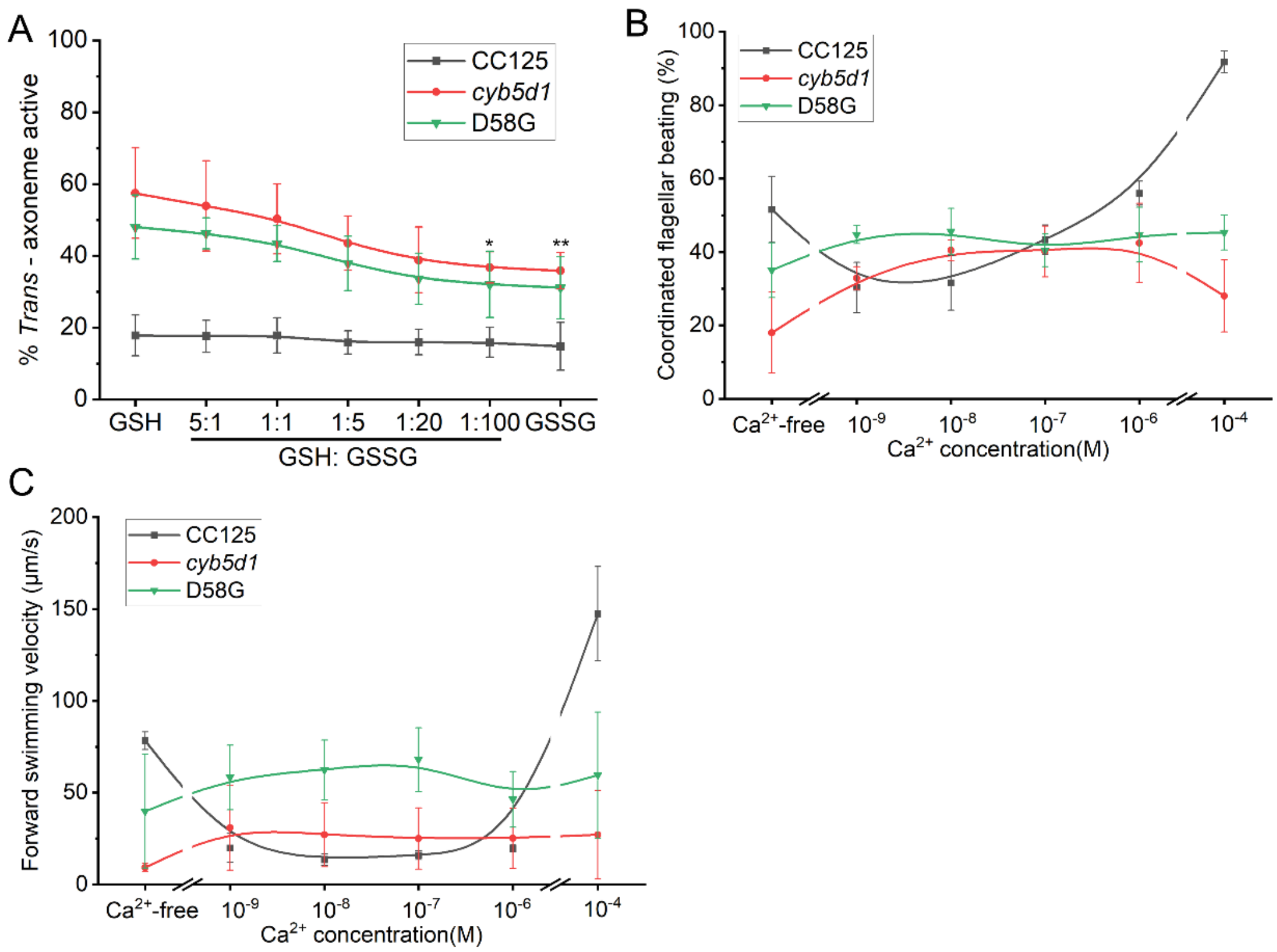

Fig. S8 Calcium regulates flagellar beating coordination and swimming velocity. (*A*) Axoneme reactivation under redox buffers (10^{-9} M free Ca^{2+}). (*B-C*) Quantification of coordinated beating and swimming velocity versus Ca^{2+} in intact cells. Data are presented as mean ± SD. Sample sizes: (*A*): N > 450 cells; (*B-C*): N > 20 cells per condition. Statistical analysis was performed using two-way ANOVA with Tukey's post-hoc test to assess the effects of strain and treatment. Statistical significance was defined as p <0.05. ****p < 0.0001.

Fig. S9

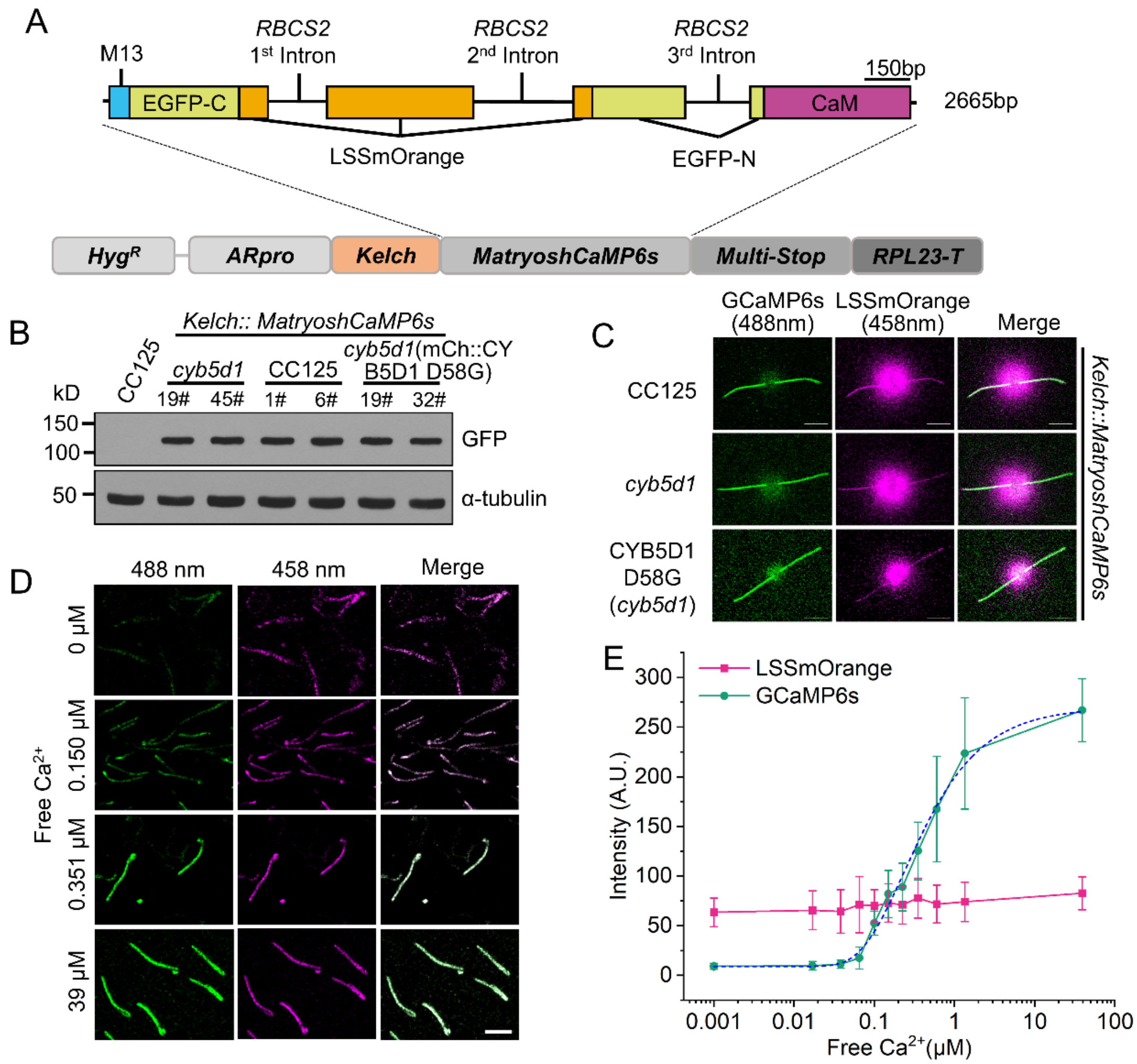

Fig. S9 Quantitative analysis of the Ca^{2+} in flagella. (*A*) Schematic diagram of the construct for expressing the calcium-sensing protein in *Chlamydomonas*. (*B-C*) Expression and localization of Kelch::MatryoshCaMP6s. (*D*) Ca^{2+}-dependent fluorescence in isolated axonemes expressing Kelch::MatryoshCaMP6s. Scale bar: 5 μm. (*E*) Semi-logarithmic plot of GCaMP6s and LSSmOrange fluorescence intensities. Data are presented as mean ± SD. The blue dashed line represents a sigmoidal curve fit to the data ($r^2 = 0.98$).

Fig. S10

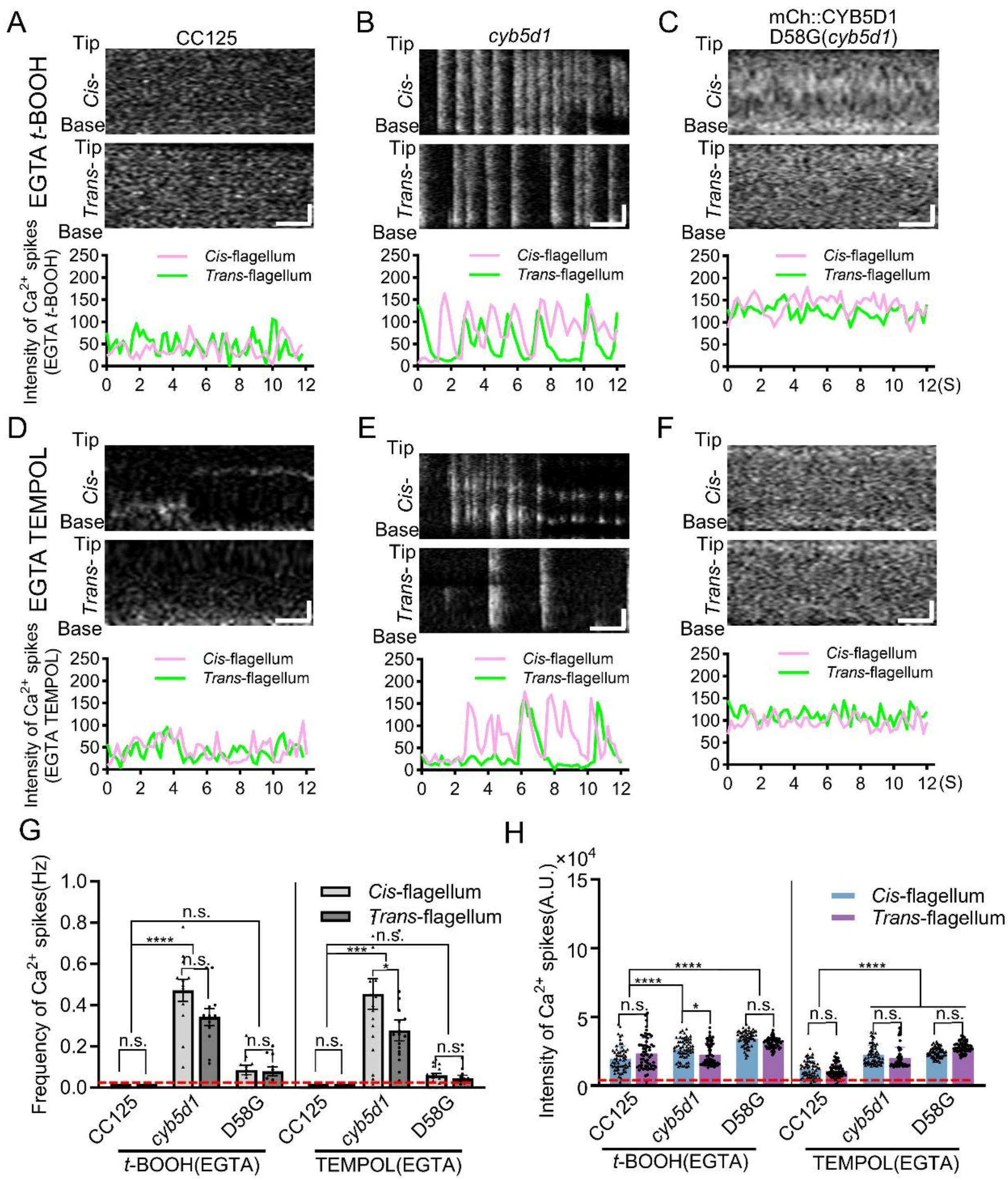

Fig. S10 Redox modulation of Ca^{2+} spikes (EGTA-buffered). (*A-F*) Ca^{2+} spikes in *cis*/*trans*-flagella after treated with *t*-BOOH/TEMPOL in the presence of 1 mM EGTA. The upper parts of each panel are the kymograph analysis, while the lower parts represent the frequency and relative abundance of Ca^{2+} spikes in the flagella. This demonstrates that redox functions upstream of calcium in ciliary regulation. Horizontal scale: 2 s; vertical scale: 2 µm. (*G-H*) Quantification of spike frequency and intensity under *t*-BOOH/TEMPOL treatment in the presence of EGTA. Data are presented as mean ± SD; n > 11 cells per strain; two-way ANOVA with Šidák's test; ****p < 0.0001; ns, not significant. Statistical significance was defined as p < 0.05.

Fig. S11

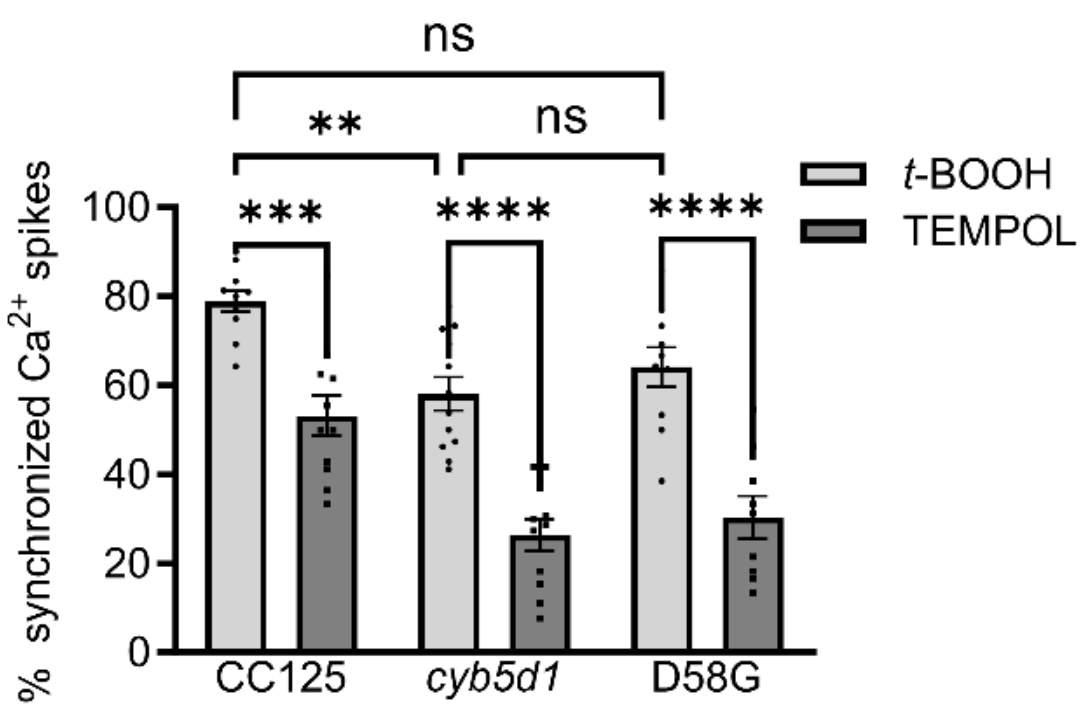

Fig. S11 Quantification of synchronized calcium spikes. Percentage of synchronized Ca^{2+} spikes in *cis*- and *trans*-flagella. The addition of the reducing agent TEMPOL reduced the proportion of synchronized Ca^{2+} spikes within the *cis*- and *trans*-flagella in all the strains (CC125, *cyb5d1*, *cyb5d1* CYB5D1(D58G)). Data are presented as mean ± SD; n > 11 cells per strain; two-way ANOVA with Šidák's test. Statistical significance was defined as p <0.05. ****p < 0.0001.

Fig. S12

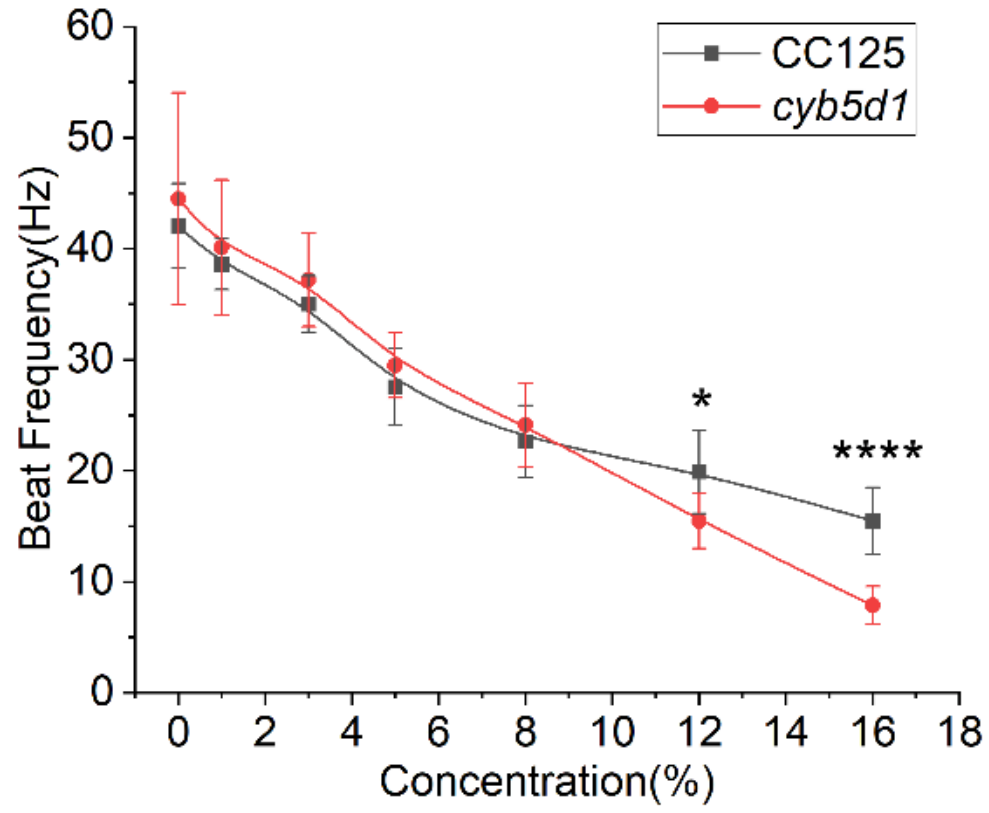

Fig. S12 Flagellar beating frequency versus viscosity. Comparison of flagellar beating frequency between CC125 and *cyb5d1* under different concentrations of Ficoll-400. 0.1% 10 μm silica was added to the solution to restrict the swimming range of the cells. Data are presented as mean ± SD. Statistical analysis was performed using two-way ANOVA with Tukey's test. ****$p < 0.0001$; n > 25 cells per condition. Statistical significance was defined as $p < 0.05$.

Fig. S13

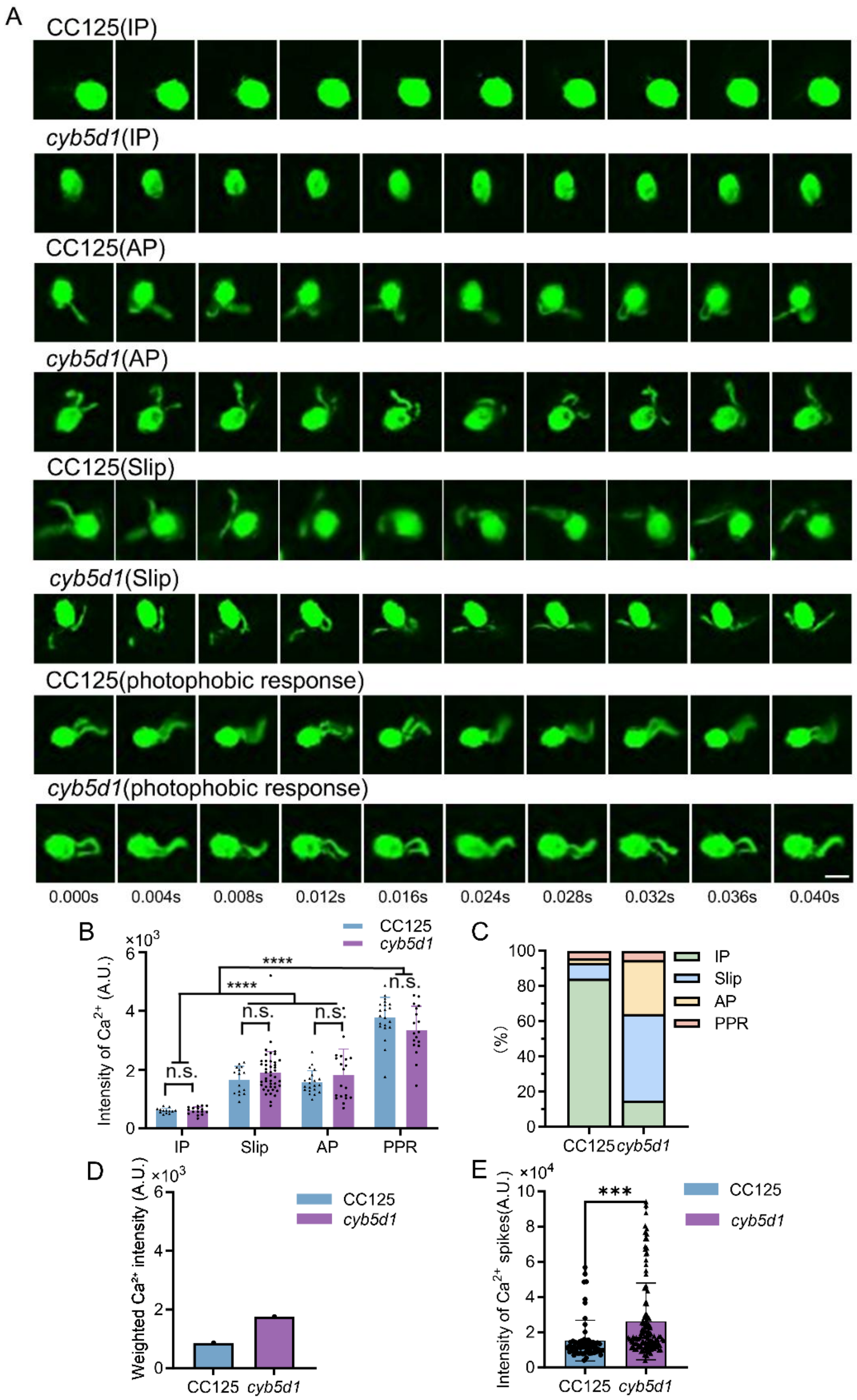

Fig. S13 Calcium intensities during distinct flagellar waveforms. (*A*) Flagellar Ca^{2+} dynamics during in-phase (IP), phase-slip (Slip), anti-phase (AP), and photophobic response (PPR) in freely swimming CC125 vs. *cyb5d1*. scale bar: 5 μm. (*B*) Quantification of Ca^{2+} spike intensities in IP,

Slip, and AP states (data from Fig. S13A). AP and Slip states exhibit higher Ca^{2+} levels than the IP state. Data are presented as mean ± SD; n > 16 cells per condition. ****$p < 0.0001$ (one-way ANOVA with Tukey's post hoc test). (*C*) Flagellar beating pattern distribution under 10% Ficoll-400 treatment in freely swimming cells. Wild-type CC125 shows > 80% in-phase (IP) beating, whereas the *cyb5d1* mutant displays a predominance of anti-phase (AP) and slip states (together ~80%). Stacked bars represent mean percentages; n > 50 cells per strain. (*D*) Weighted average Ca^{2+} intensity in wild-type and *cyb5d1*. Weighted intensity was calculated as the sum of the beating-state fraction × state-specific Ca^{2+} intensity including all behavioral states; state fractions and state-specific intensities are from Figs. S13C and S13B, respectively. (*E*) Mean Ca^{2+} spike intensity in agarose-immobilized cells (untreated; data from Fig. 5H). The *cyb5d1* mutant shows approximately twice the Ca^{2+} intensity (~26,000 a.u.) of wild-type CC125 (~15,000 a.u.). Data are presented as mean ± SD; n > 16 cells per strain. Statistical significance was assessed by unpaired two-tailed *t*-test; ($\alpha = 0.05$), ***$p < 0.001$.

Fig. S14

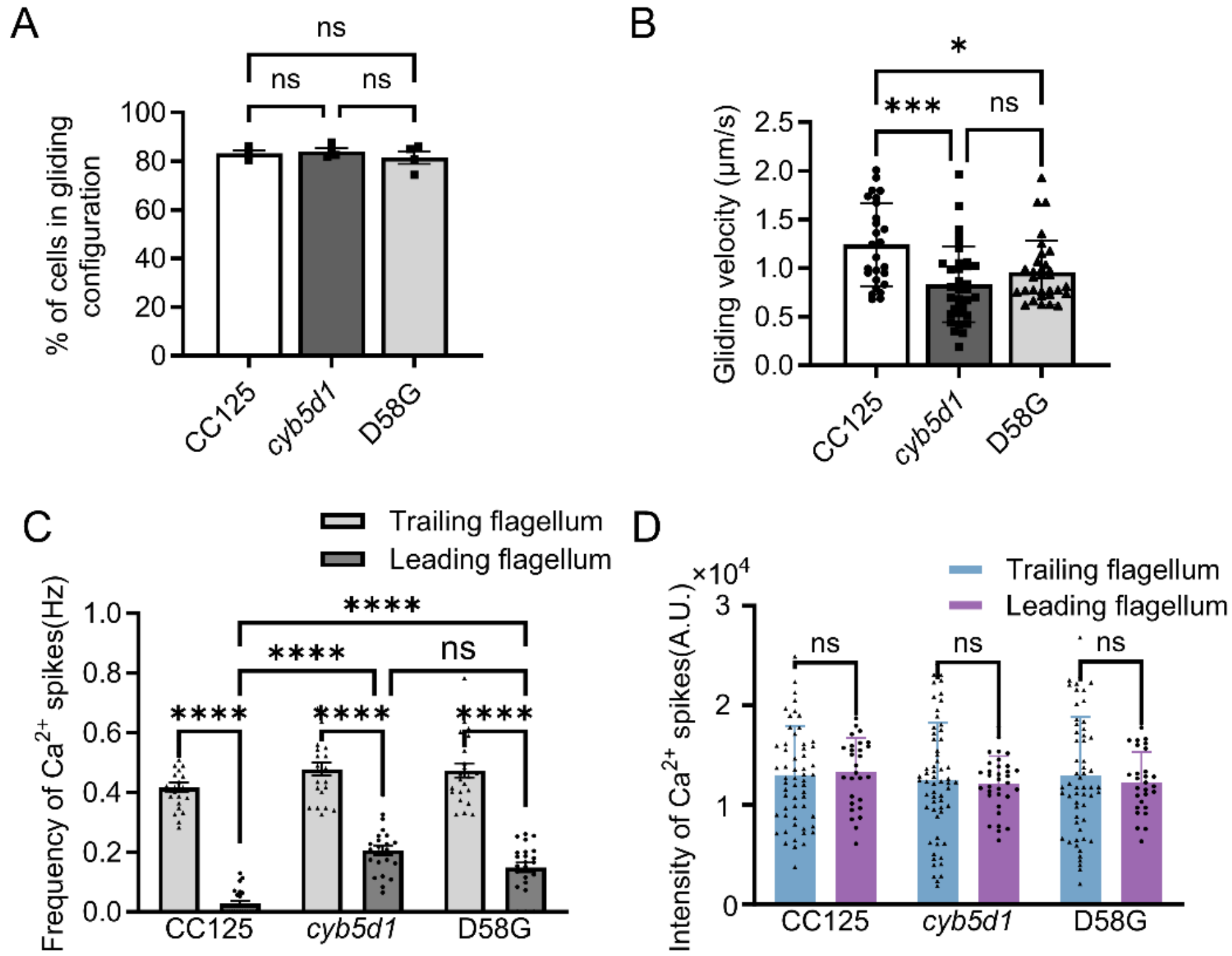

Fig. S14 The gliding configuration, gliding velocity and the flagellar Ca^{2+} dynamics during gliding in *cyb5d1* and *cyb5d1(D58G)* mutants. (*A*) Percentage of cells adopting the gliding configuration in wild-type CC125, *cyb5d1*, and *cyb5d1* CYB5D1(D58G) strains (n = 4 independent experiments, > 200 cells per strain; one-way ANOVA with Tukey's test; ns, not significant). (*B*) Gliding velocity of individual cells in wild-type CC125, *cyb5d1*, and *cyb5d1* CYB5D1(D58G) strains. Data are presented as mean ± SD; n > 20 cells per strain; one-way ANOVA with Tukey's test; ***$p < 0.001$. (*C*) The frequency of Ca^{2+} spike in the trailing flagellum and leading flagellum during gliding in wild-type CC125, *cyb5d1*, and *cyb5d1* CYB5D1(D58G) strains. Data are presented as mean ± SD; n > 20 cells per strain; two-way ANOVA with Šidák's test; ****$p < 0.0001$; ns, not significant. (*D*) The intensity of Ca^{2+} spike in the trailing flagellum and leading flagellum during gliding in wild-type CC125, *cyb5d1*, and *cyb5d1* CYB5D1(D58G) strains. Data are presented as mean ± SD; n > 25 cells per strain; two-way ANOVA with Šidák's test; ns, not significant. Statistical significance was defined as $p < 0.05$.

Table S1. Formula of redox buffers

Buffer No.	DTT_{ox} (mM)	DTT_{red} (mM)	Redox potential (mV)
1	20	8	-342.2
2	20	2	-324.4
3	20	0.8	-312.7
4	20	0.4	-303.8
5	20	0.2	-294.9
6	20	0.08	-283.1
7	20	0.04	-274.2
8	20	0.02	-265.3
9	20	0.008	-253.5
10	20	0.004	-244.6
11	20	0.002	-235.7
12	20	0.001	-226.8

Final concentrations in the solutions. Components other than DTT: 30 mM Hepes, 5 mM MgSO4, 50 mM potassium acetate, 1% PEG (average Mw=20,000). The pH of all buffers was adjusted to pH 7.4.

Movie S1. Confocal video showing the flagellar Ca^{2+} dynamics of CC125, *cyb5d1* and *cyb5d1* CYB5D1(D58G).

Movie S2. Confocal video showing the flagellar Ca^{2+} dynamics of CC125, *cyb5d1* and *cyb5d1* CYB5D1(D58G) under 1 mM EGTA treatment.

Movie S3. Confocal video showing the flagellar Ca^{2+} dynamics of CC125, *cyb5d1* and *cyb5d1* CYB5D1(D58G) under 0.4 mM oxidant *t*-BOOH treatment.

Movie S4. Confocal video showing the flagellar Ca^{2+} dynamics of CC125, *cyb5d1* and *cyb5d1* CYB5D1(D58G) under 40 mM reductant TEMPOL treatment.

Movie S5. Confocal video showing the flagellar Ca^{2+} dynamics of CC125, *cyb5d1* and *cyb5d1* CYB5D1(D58G) under treatment with 0.4 mM oxidant *t*-BOOH in the presence of 1 mM EGTA.

Movie S6. Confocal video showing the flagellar Ca^{2+} dynamics of CC125, *cyb5d1* and *cyb5d1* CYB5D1(D58G) under treatment with 40 mM reductant TEMPOL in the presence of 1 mM EGTA.

Movie S7. HS211 flagellar beat frequency example.

SI References

1. L. Zhao *et al.*, Heme-binding protein CYB5D1 is a radial spoke component required for coordinated ciliary beating. *Proc. Natl. Acad. Sci. U. S. A.* **118** (2021).
2. X. Cheng *et al.*, Building a multipurpose insertional mutant library for forward and reverse genetics in *Chlamydomonas*. *Plant Methods* **13**, 36 (2017).
3. B. Morgan *et al.*, Real-time monitoring of basal H_2O_2 levels with peroxiredoxin-based probes. *Nat. Chem. Biol.* **12**, 437-443 (2016).
4. C. Ast *et al.*, Ratiometric Matryoshka biosensors from a nested cassette of green- and orange-emitting fluorescent proteins. *Nat. Commun.* **8**, 431 (2017).
5. T. Baier, J. Wichmann, O. Kruse, K. J. Lauersen, Intron-containing algal transgenes mediate efficient recombinant gene expression in the green microalga *Chlamydomonas reinhardtii*. *Nucleic. Acids. Res.* **46**, 6909-6919 (2018).
6. J. Niemeyer, D. Scheuring, J. Oestreicher, B. Morgan, M. Schroda, Real-time monitoring of subcellular H_2O_2 distribution in *Chlamydomonas reinhardtii*. *Plant Cell* **33**, 2935-2949 (2021).
7. K. Huang *et al.*, Function and dynamics of PKD2 in *Chlamydomonas reinhardtii* flagella. *J. Cell Biol.* **179**, 501-514 (2007).
8. R. Kamiya, G. B. Witman, Submicromolar levels of calcium control the balance of beating between the two flagella in demembranated models of *Chlamydomonas*. *J Cell Biol* **98**, 97-107 (1984).
9. P. Collingridge, C. Brownlee, G. L. Wheeler, Compartmentalized calcium signaling in cilia regulates intraflagellar transport. *Curr. Biol.* **23**, 2311-2318 (2013).
10. Cold Spring Harb Protoc 2015; 2015(5): pdb.rec079624. doi:10.1101/pdb.rec079624.
11. H. Ishikawa, J. Moore, D. R. Diener, M. Delling, W. F. Marshall, Testing the ion-current model for flagellar length sensing and IFT regulation. *Elife* **12** (2023).
12. K. Sugiura, Y. Nishimaki, M. Owa, T. Hisabori, K. I. Wakabayashi, Assessment of the flagellar redox potential in *Chlamydomonas reinhardtii* using a redox-sensitive fluorescent protein, Oba-Qc. *Biochem. Biophys. Res. Commun.* **503**, 2083-2088 (2018).
13. A. J. Meyer, T. P. Dick, Fluorescent protein-based redox probes. *Antioxid Redox Signal* **13**, 621-650 (2010).
14. I. Kimura *et al.*, Neurotrophic activity of neudesin, a novel extracellular heme-binding protein, is dependent on the binding of heme to its cytochrome b5-like heme/steroid-binding domain. *J. Bio.l Chem.* **283**, 4323-4331 (2008).
